# Supplementary figures and images for: Using de novo assembly to identify structural variation of eight complex immune system gene regions
Source: PLoS Comput Biol. 2021 Aug 3;17(8):e1009254. doi: 10.1371/journal.pcbi.1009254 (PMC8363018; doi:10.1371/journal.pcbi.1009254)

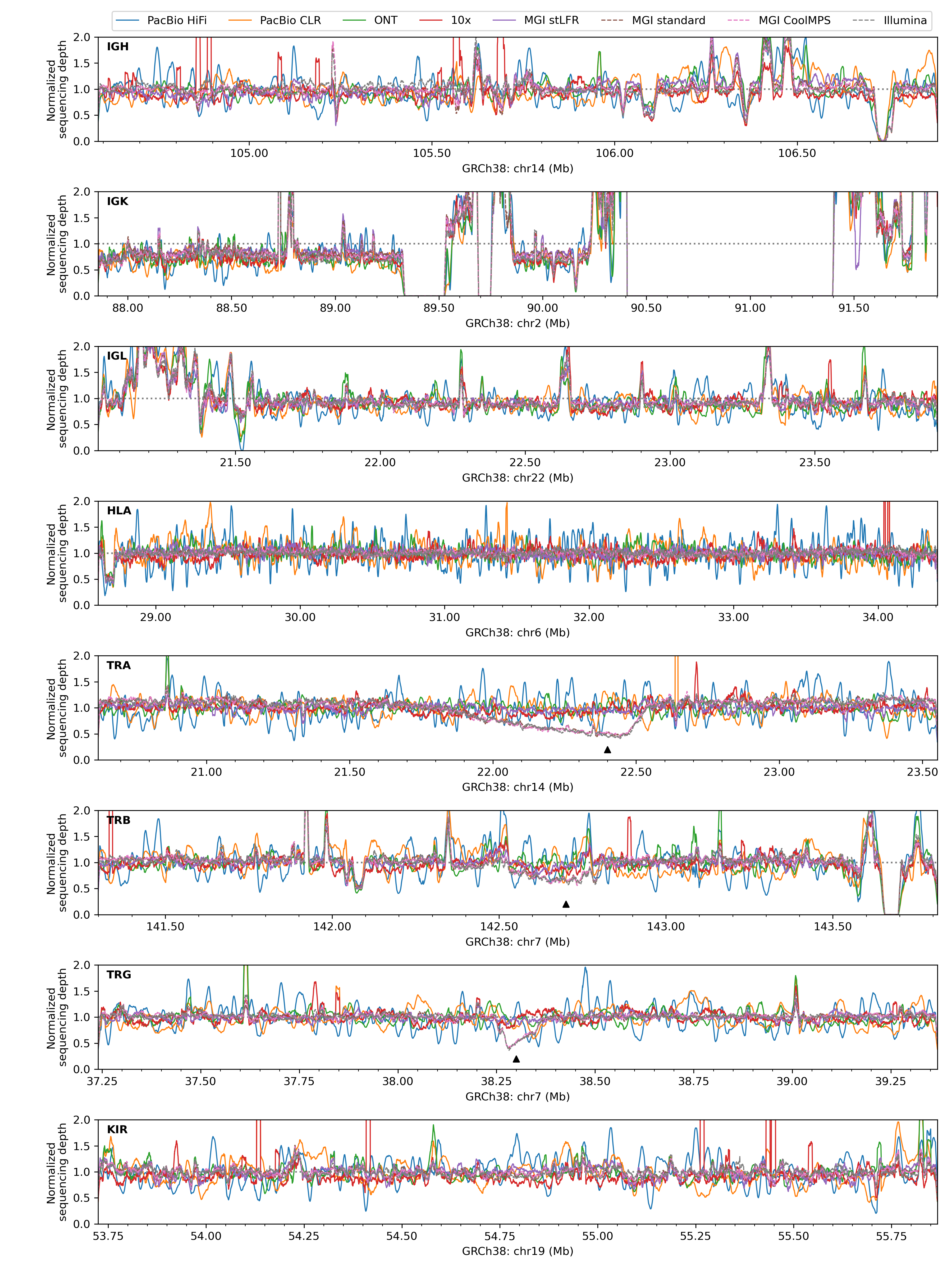

Supplement: S1 Fig — For each sequencing platform (colored lines, with platforms as in S1 Table), the plot shows the depth of coverage of reads aligned to GRCh38 across the eight selected regions (Table 1). Depths are normalized by the average depth across each region for each dataset. Areas with apparent systematic lower depth in the TRA, TRB and TRG regions are highlighted with black triangles. Datasets generated with DNA from CD14+ monocyte and PMBC are denoted with solid and dashed lines, respectively. Decrease of sequencing depths is not identified in immunoglobulin regions, presumably due to the relatively low fraction (5–15%) of B cells in PBMC. Despite γδ T cells being rarer than α/β T cells, the coverage drop around T cell receptor γ genes can be explained by the fact that the γ locus is known to undergo rearrangement in most α/β T cells [65], and the drop around T cell receptor δ genes can be explained by the fact that any rearrangement at the α locus leads to the loss of the δ locus.[66] (PNG) [file pcbi.1009254.s001.png]

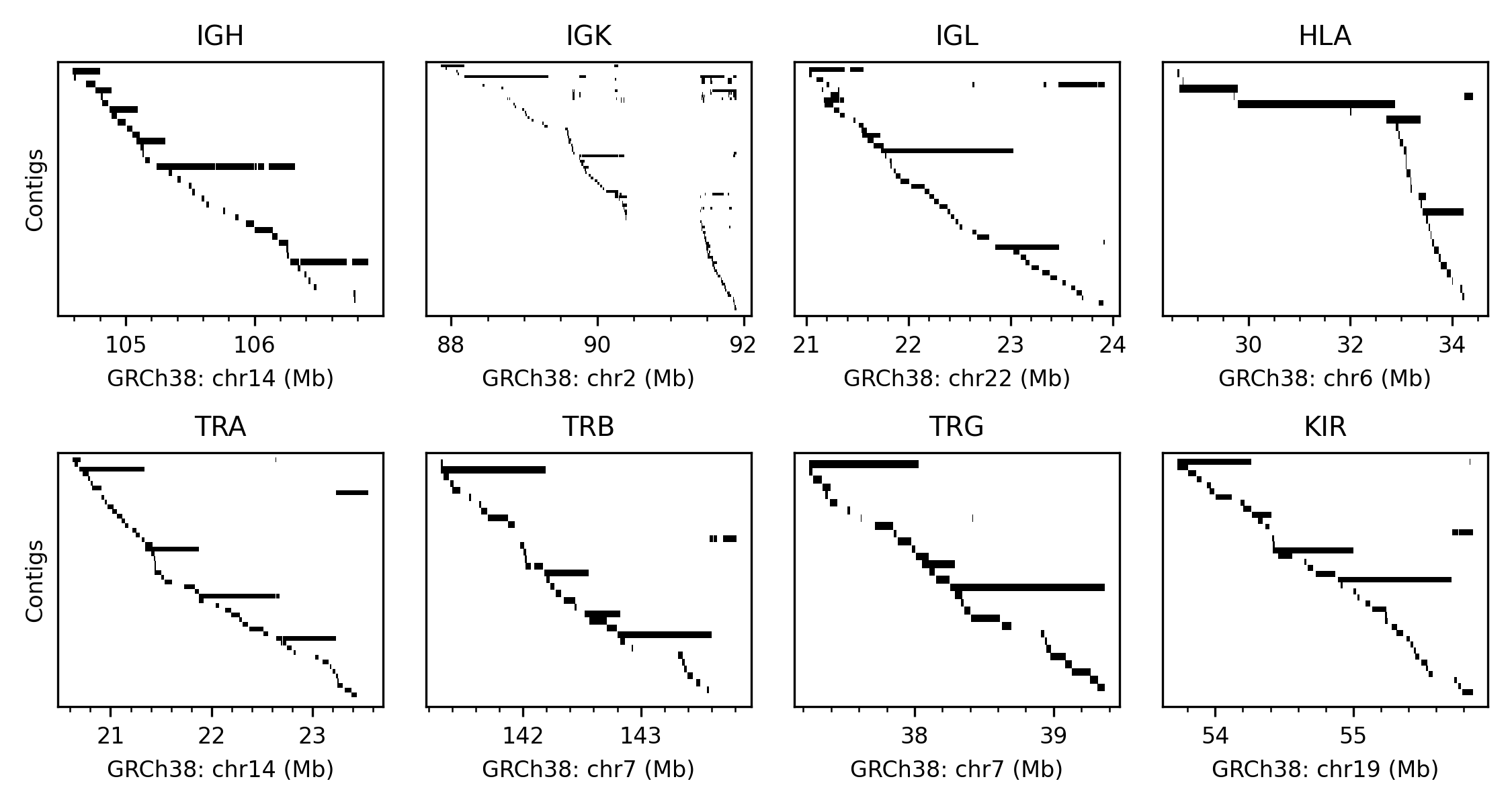

Supplement: S2 Fig — Each row represents one local contig extracted from the draft whole-genome assembly (Fig 1A) by alignment to GRCh38 using minimap2 (Methods). The x axis reflects GRCh38 coordinates across each of the selected regions (Table 1). (PNG) [file pcbi.1009254.s002.png]

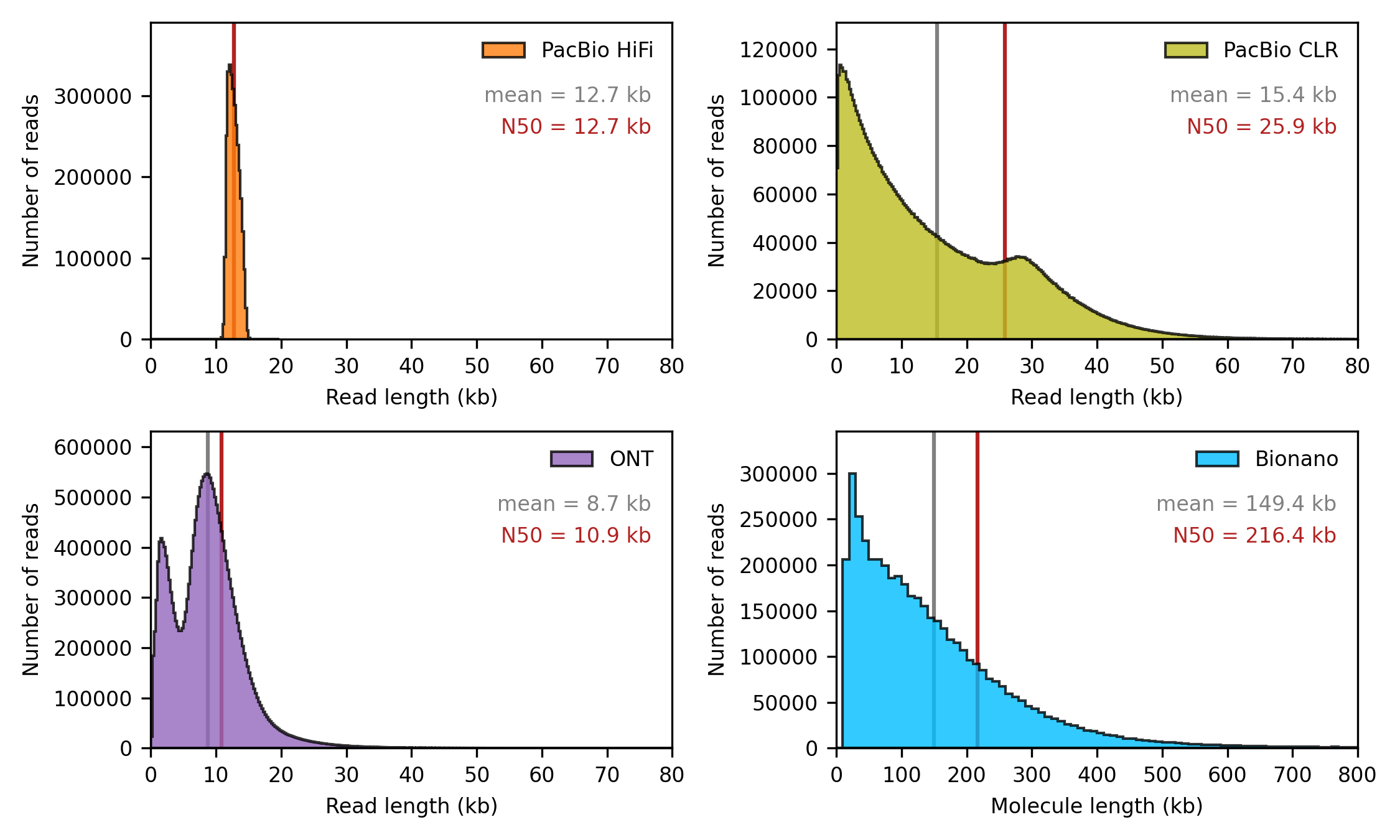

Supplement: S3 Fig — Red and grey vertical lines denote the N50 (i.e. the maximal length such that reads/molecules longer than this length cumulatively account for at least 50% of the total length of reads/molecules in the dataset) and mean read/molecule length for each dataset, respectively. (PNG) [file pcbi.1009254.s003.png]

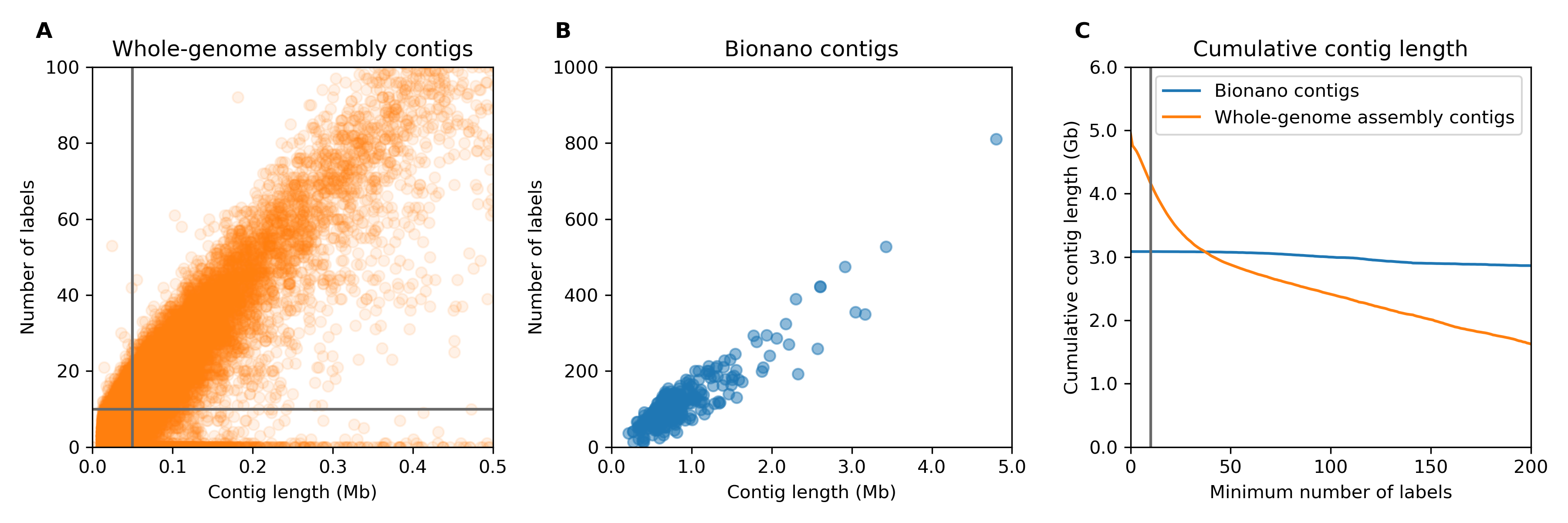

Supplement: S4 Fig — (A,B) Number of DLE-1 labels (y axis) plotted against contig length (x axis) for draft whole-genome assembly contigs (panel A) and Bionano contigs (panel B). For reference, gray vertical and horizontal lines in panel A denote 50 kb length and 10 DLE-1 labels, respectively. (C) Cumulative length of contigs (y axis) containing at least the given number of DLE-1 labels (x axis) is shown for whole-genome assembly contigs (orange) and Bionano contigs (blue). For reference, the gray vertical line denotes 10 DLE-1 labels. (PNG) [file pcbi.1009254.s004.png]

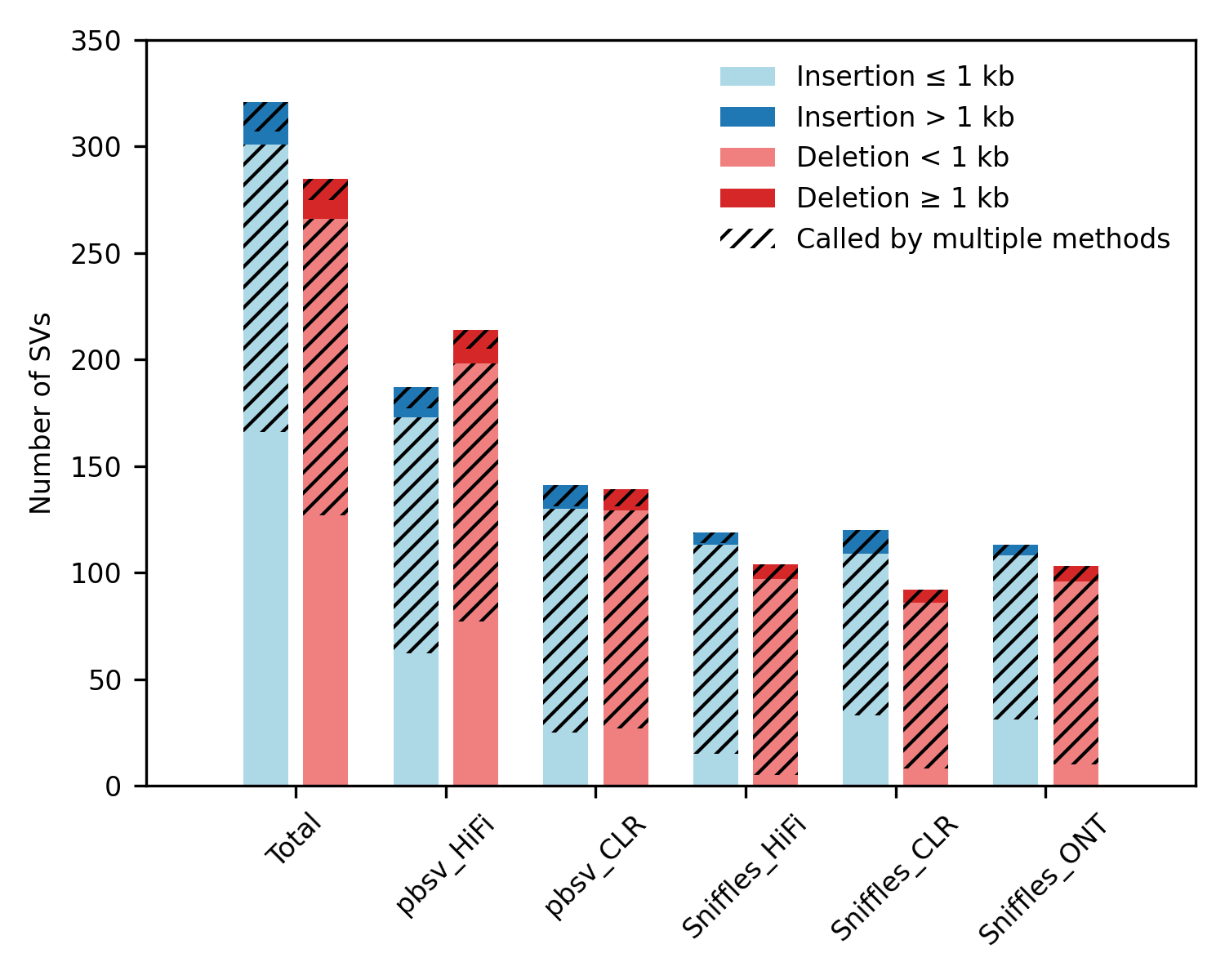

Supplement: S5 Fig — Bars show the number of insertions (blue) and number of deletions (red) identified by each combination of method and sequencing data (x axis) after aligning reads to the HV31 assembly as described in main text and Methods. SVs are classified as insertions or deletions according to whether the alternative haplotype is longer or shorter than the HV31 haplotype. For comparison, the number of SVs called by multiple methods, as identified by SVanalyzer, is indicated by shading. (PNG) [file pcbi.1009254.s005.png]

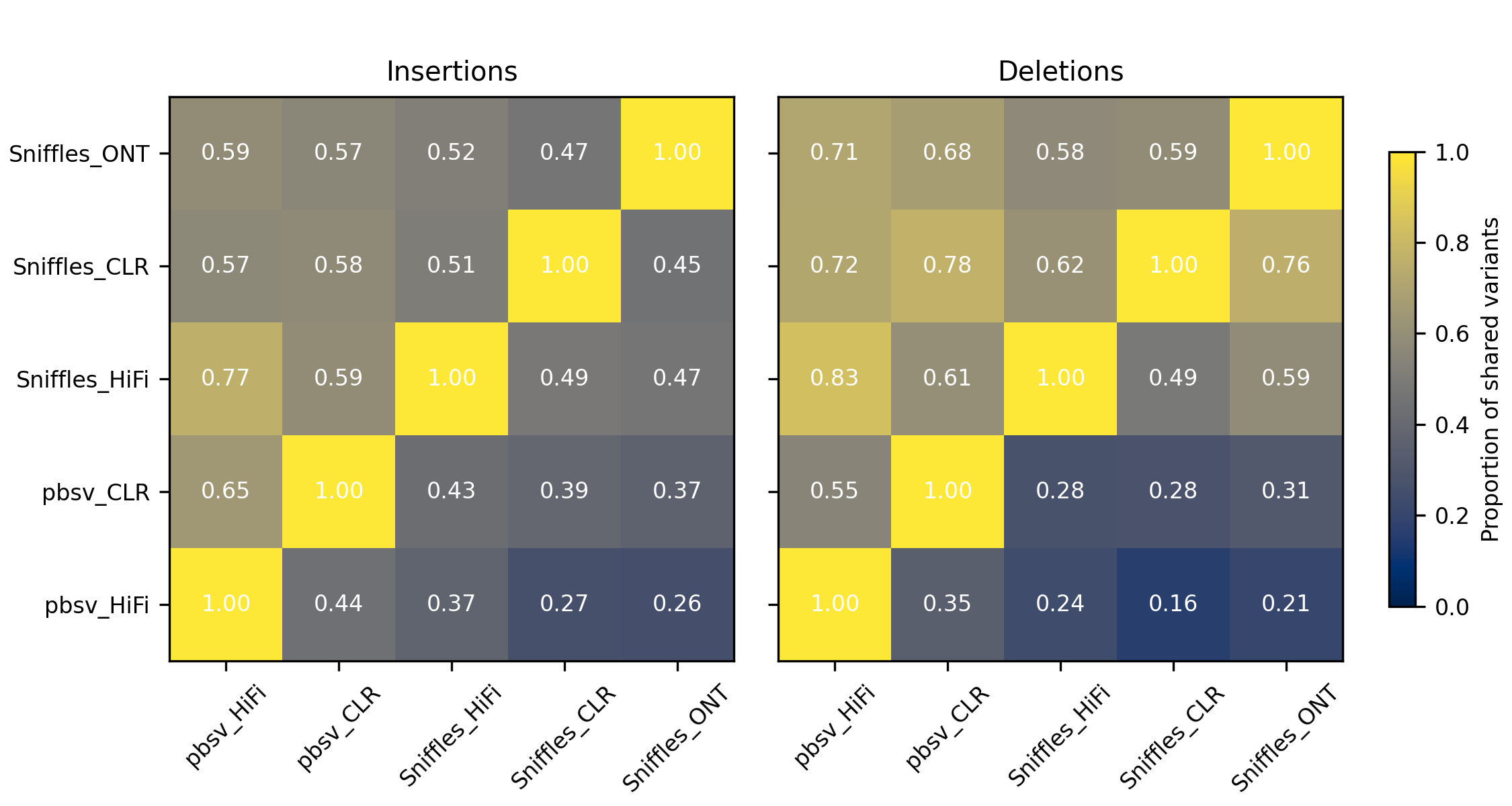

Supplement: S6 Fig — Each row shows the fraction of variants called by the corresponding method (y axis), that are also called by the method in the relevant column (x axis). Concordance of SVs is as determined by SVanalyzer. (PNG) [file pcbi.1009254.s006.png]

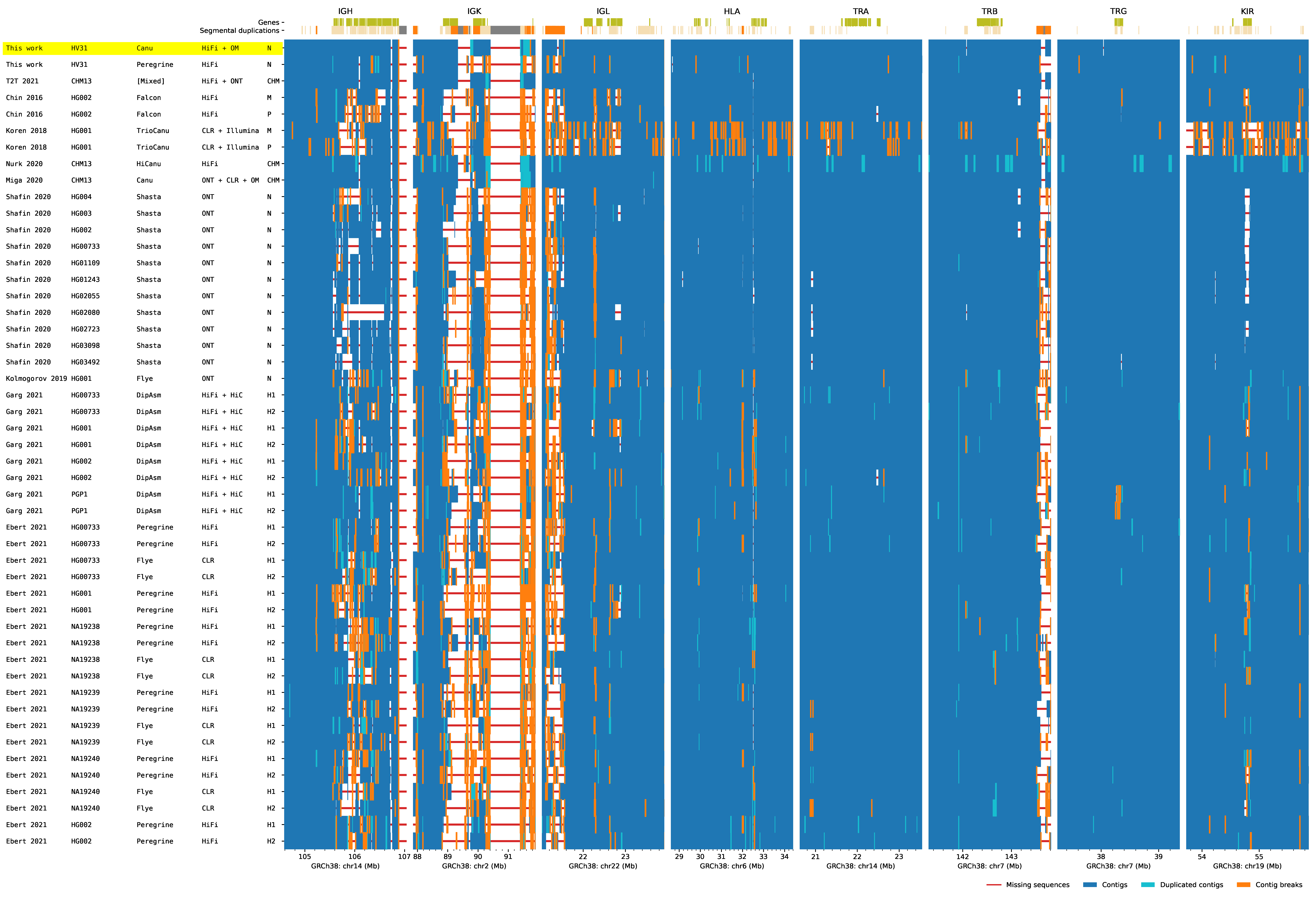

Supplement: S7 Fig — Each row visualizes the alignment pattern of the corresponding assembly with GRCh38 as the reference. Duplicate contigs (i.e. shorter contigs that align within the span of a longer contig) and contig breaks (identified as endpoints of non-duplicate contigs) are shown in cyan and orange respectively, with regions of the GRCh38 reference that are not covered by the aligned assembly contigs denoted by red lines, according to the legend. Each assembly is labeled in the following order: publication, sample, key algorithms, key technology (with OM denoting optical mapping and Hi-C denoting the Hi-C chromosome conformation capture approach) and haplotype (N, not haplotype-resolved; M, maternal haplotype; P, paternal haplotype; H1, haplotype 1; H2, haplotype 2; CHM, complete hydatidiform mole). Relevant publications are: T2T 2021 [32], Chin 2016 [49], Koren 2018 [67], Nurk 2020 [31], Miga 2020 [29], Shafin 2020 [68], Kolmogorov [69], Garg 2021 [16], Ebert 2021 [17]. Genes and segmental duplications are annotated above as in Fig 2. (PNG) [file pcbi.1009254.s007.png]

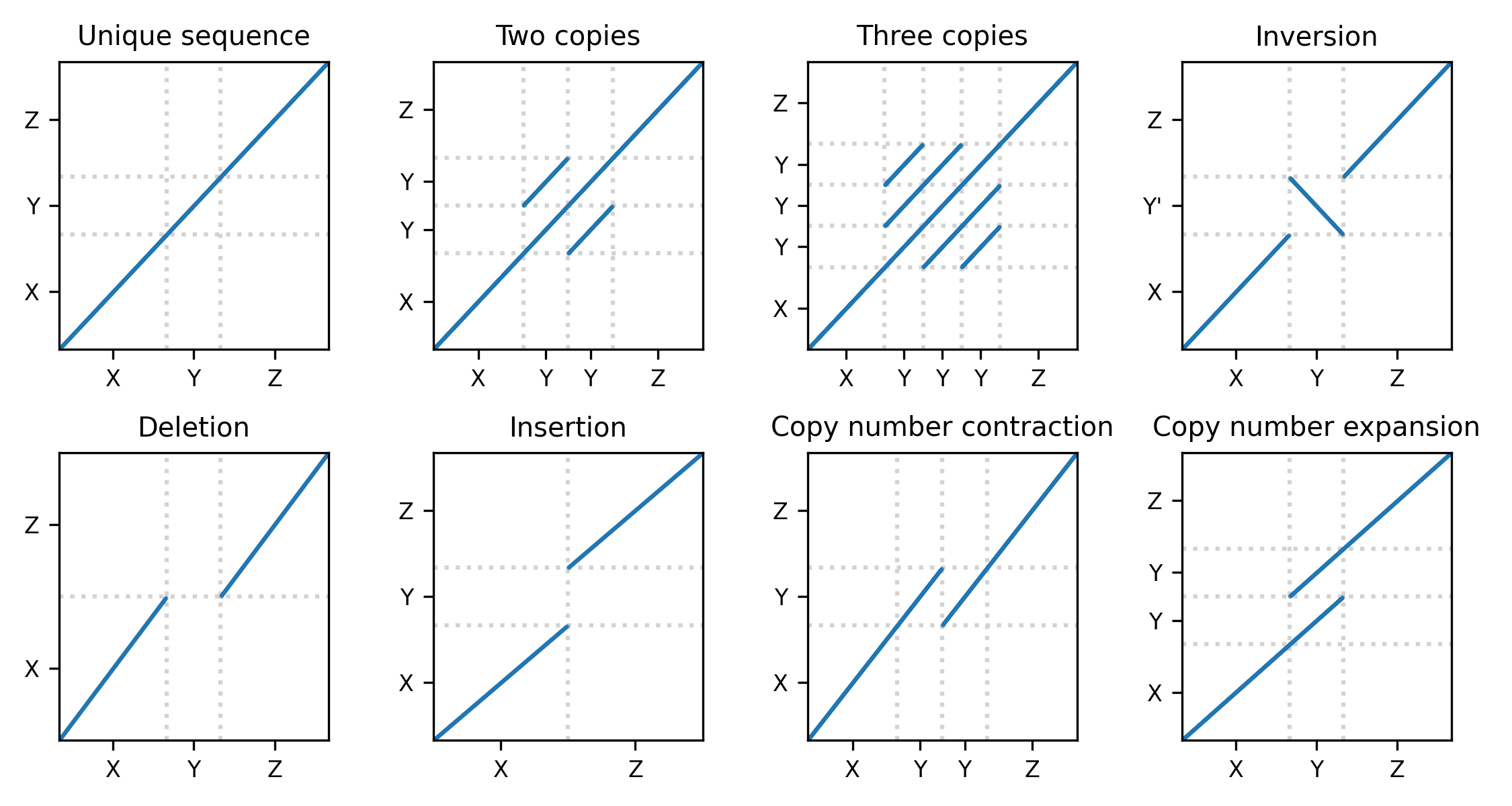

Supplement: S8 Fig — Each panel shows a schematic of the expected pattern visualized on the k-mer sharing plots, given the pattern of sequence duplication or structural variant indicated by the panel label. For clarity, specific sequences are labelled as follows: X, Y and Z denote sequence fragments that are different from each other, and Y’ denotes the reverse complement of Y. In each panel, the reference sequence is depicted on the x axis and the alternate sequence is depicted on the Y axis. The size of each structural variant can be estimated from the distance between relevant breakpoints on the plot. (PNG) [file pcbi.1009254.s008.png]

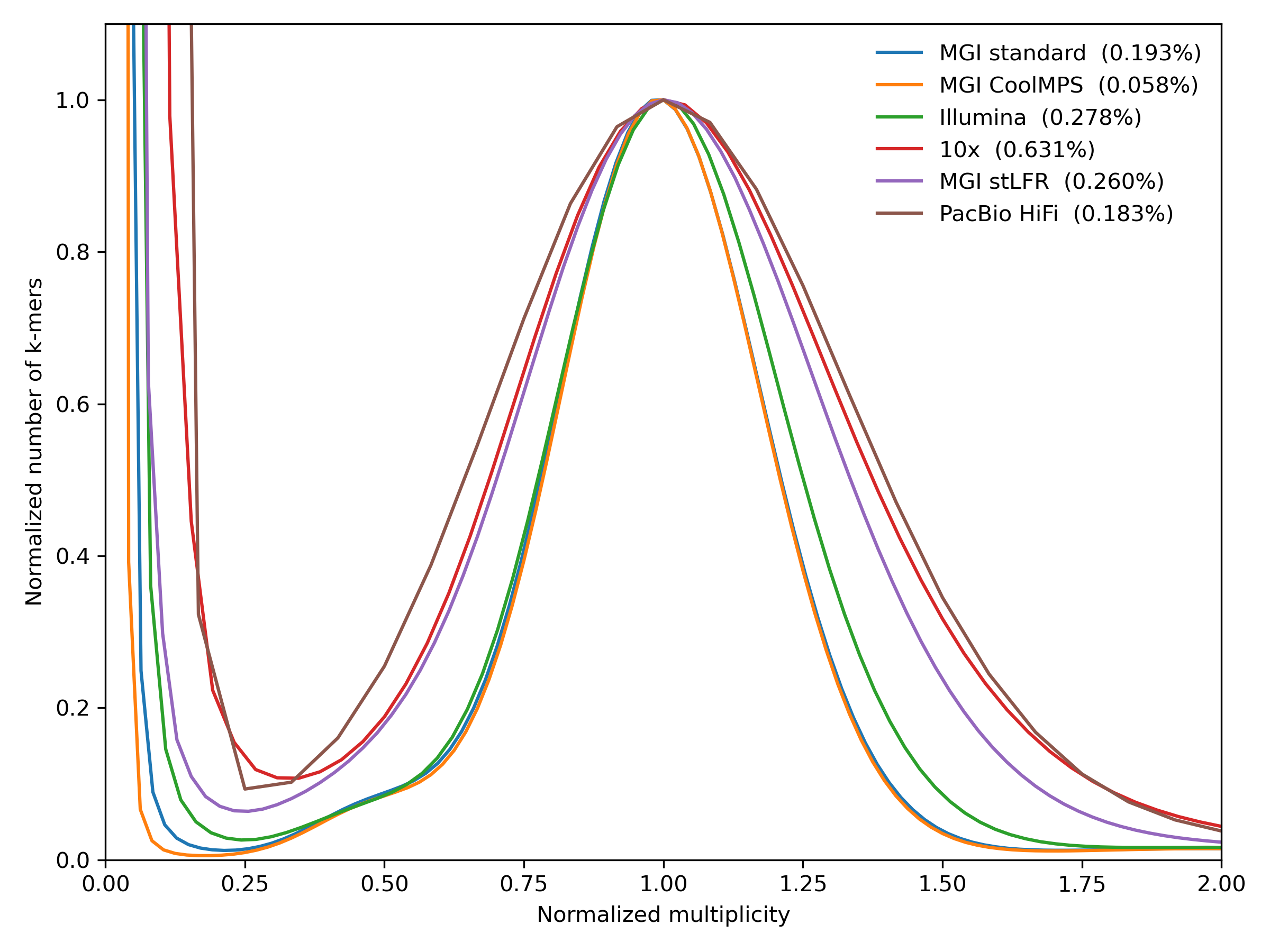

Supplement: S9 Fig — The histogram of k-mer (k = 22) multiplicity in each dataset is shown, after scaling multiplicity values (x axis) and k-mer numbers (y axis) so that the peak of unique homozygous k-mers in each dataset overlap at x = 1, y = 1. Numbers in brackets show the estimated per-base error rate of each dataset as estimated using GenomeScope [35]. (PNG) [file pcbi.1009254.s009.png]

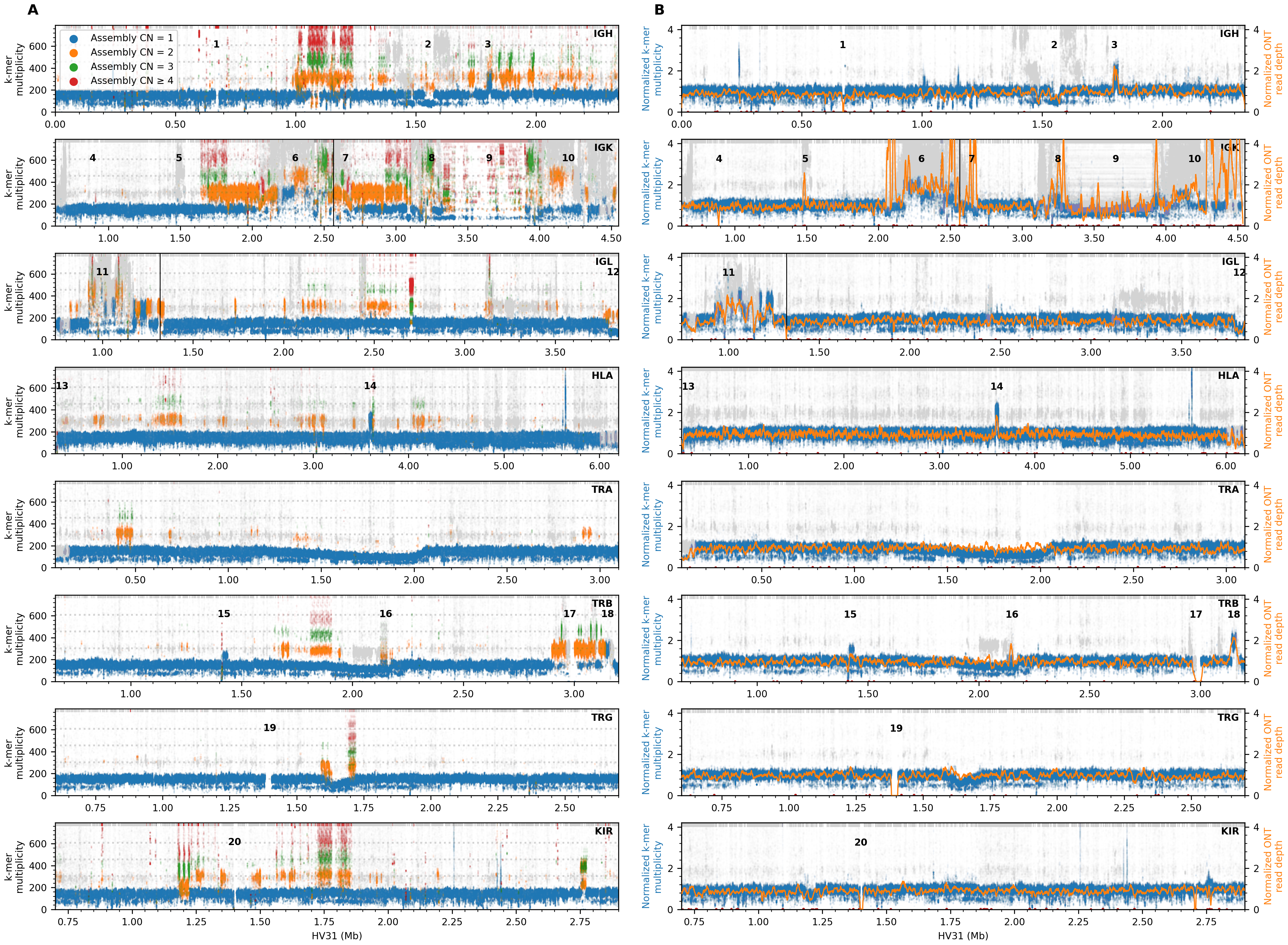

Supplement: S10 Fig — (A) For each k-mer (k = 31) that appears in the HV31 assembly, the multiplicity of that k-mer in the validation dataset (y axis) is plotted against the position of that k-mer (x axis), colored by the copy number of that k-mer in the HV31 assembly as shown in the legend. (B) the normalized k-mer multiplicity (y axis), defined as ratio of validation k-mer multiplicity to assembly k-mer multiplicity computed using the values shown in (A), plotted against the position of that k-mer (x axis). Values are further normalized by dividing by the peak multiplicity of unique homozygous k-mers as shown in Fig 3A, such that these kmers are expected to lie near y = 1. k-mers that found both inside and outside the given regions are considered noninformative and are shown in gray. Orange lines show ONT read coverage depth normalized to the genome-wide average coverage depth (63×). (PNG) [file pcbi.1009254.s010.png]

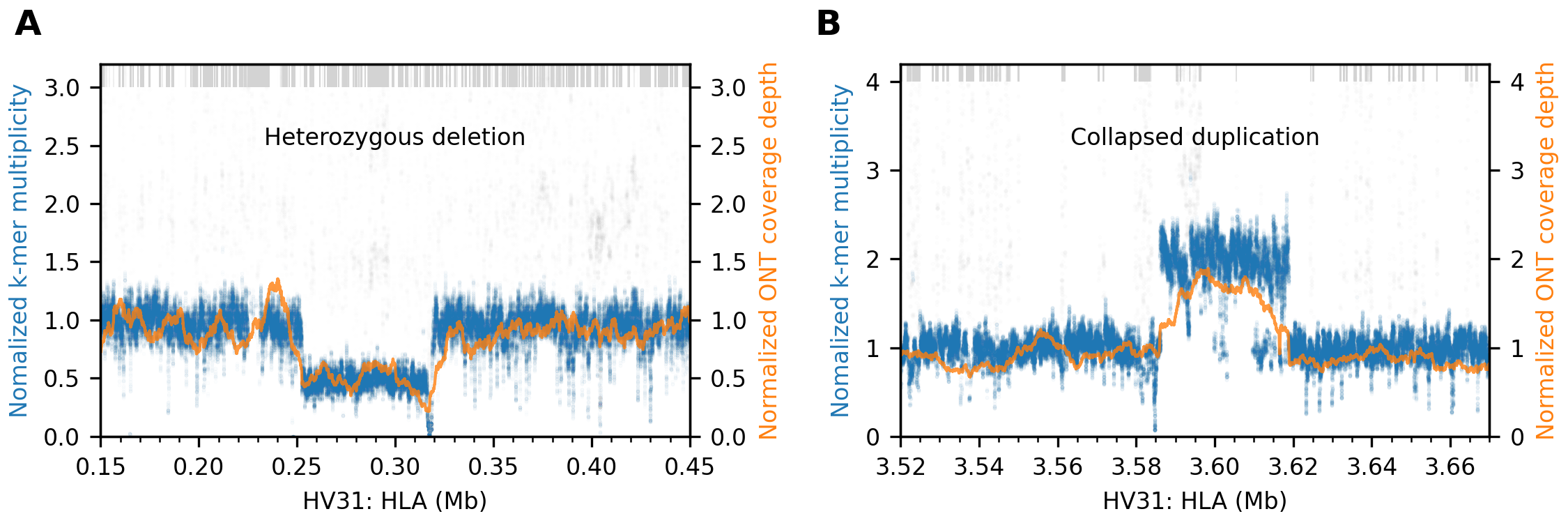

Supplement: S11 Fig — (A) A 63.9 kb heterozygous deletion in the HLA locus is revealed by reduced ONT coverage depth (orange) and validation k-mer multiplicity (k = 31; blue) appearing at normalized multiplicity close to 0.5. (B) A collapsed duplication in the HLA locus is revealed by elevated ONT coverage depth (orange) and validation k-mer multiplicity (blue) appearing at normalized multiplicity close to 2. k-mers that found both inside and outside the IGH region are considered noninformative and are shown in gray. In (A) and (B), k-mers with multiplicity beyond the axis limits are stacked at the top of the plots. (PNG) [file pcbi.1009254.s011.png]

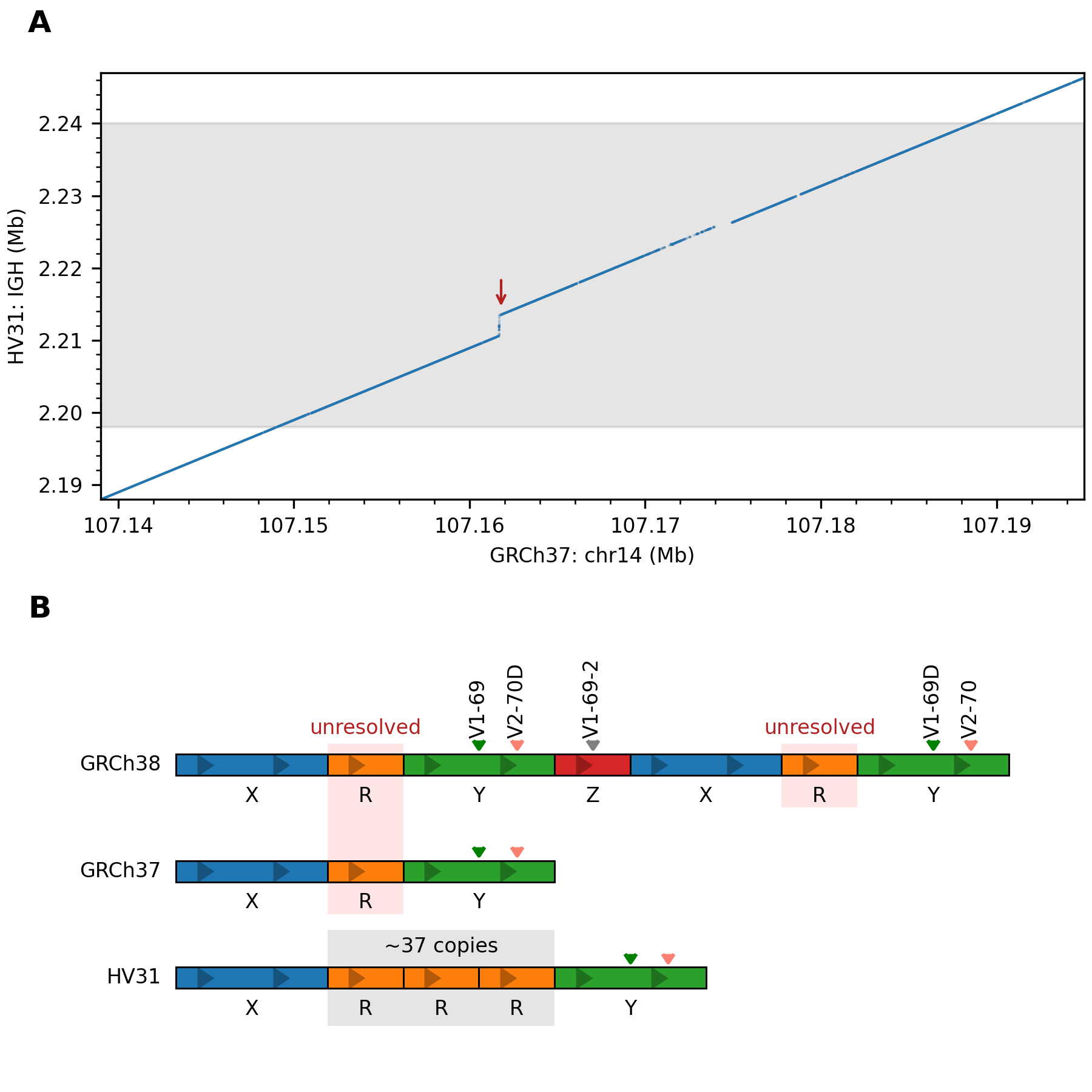

Supplement: S12 Fig — (A) k-mer sharing plot (k = 50) comparing GRCh37 and GRCh38 in the IGH region. Similar to HV31, GRCh37 has only one copy of IGHV1-69 and IGHV2-70 genes. The unresolved repeats are highlighted with red arrows. Gray shade marks the position of the 45 kb CNV in HV31 relative to GRCh38 (see Fig 3A). (B) Schematic representation of GRCh38, GRCh37 and HV31 near IGHV1-69 and IGHV2-70 genes. Fragment R denotes the unresolved duplication which was assembled in HV31. (PNG) [file pcbi.1009254.s012.png]

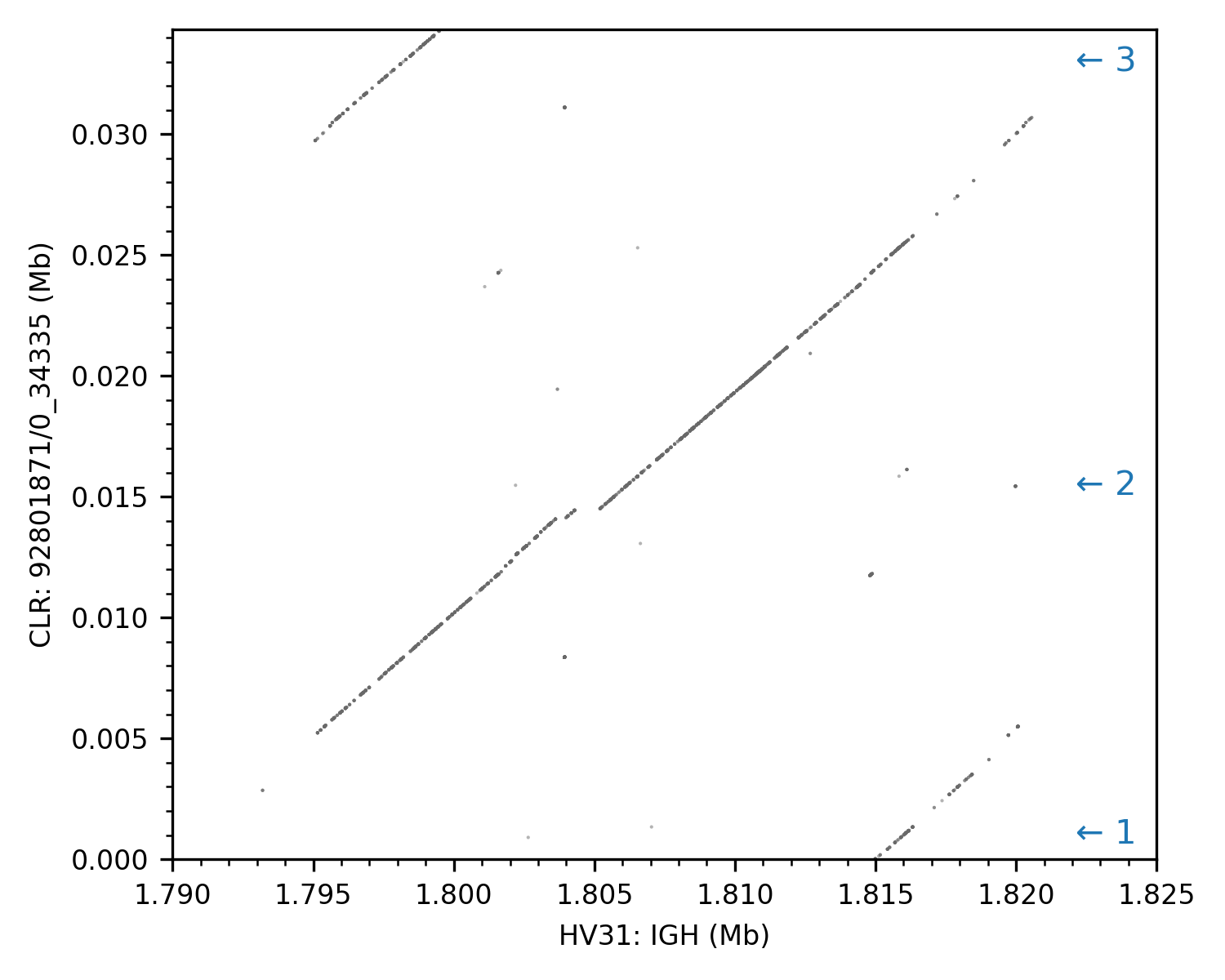

Supplement: S13 Fig — k-mer sharing plot (k = 20) comparing the CLR read with ID 92801871/034335 (y axis) with the HV31 assembly (x axis). The read is consistent with the presence of a three-copy unassembled haplotype as described in main text and Fig 5D. Each copy of the repeat unit is annotated with a number and an arrow for clarity. (PNG) [file pcbi.1009254.s013.png]

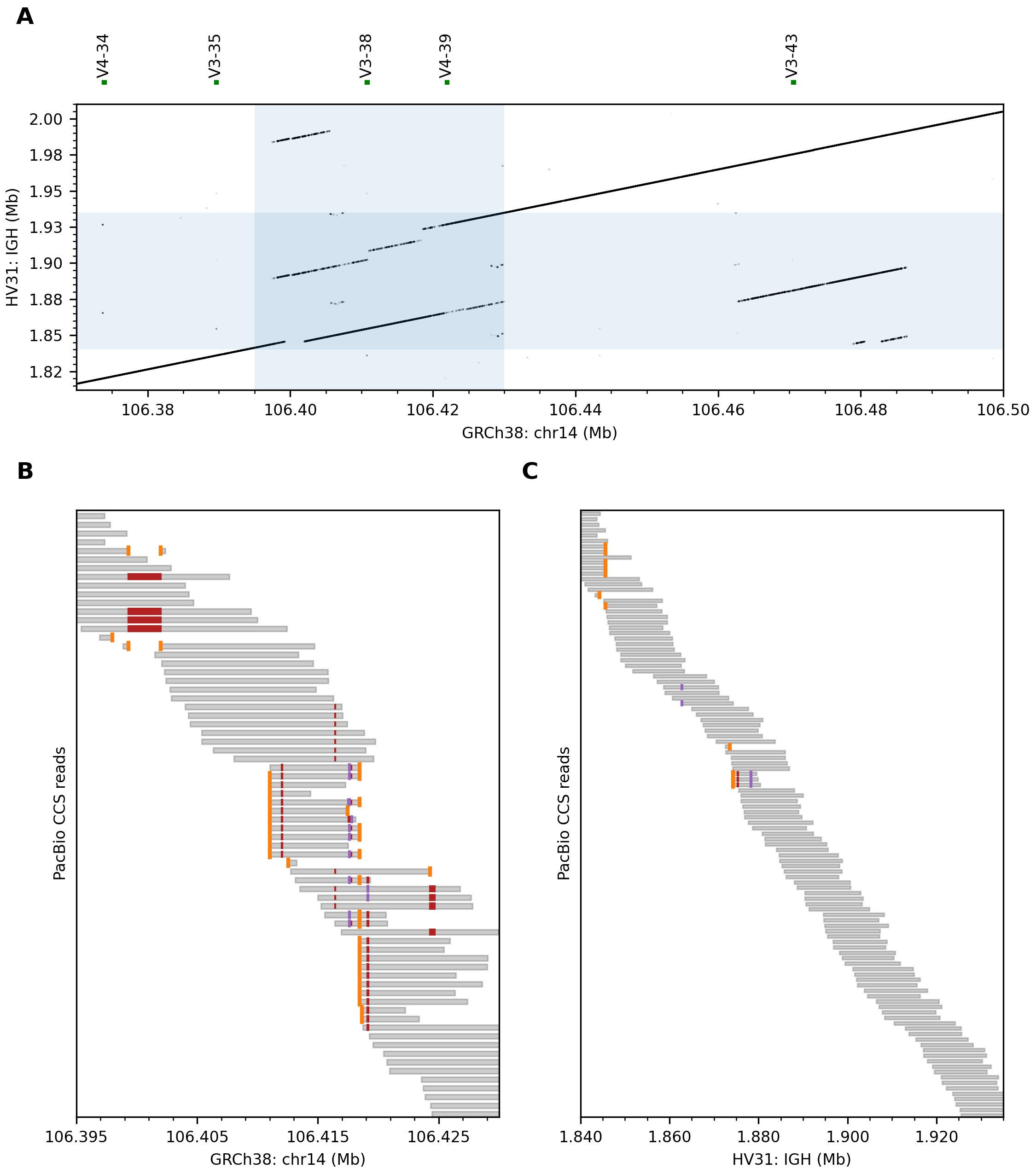

Supplement: S14 Fig — (A) k-mer sharing plot (k = 50) comparing the HV31 assembly (y axis) with GRCh38 (x axis), detailing the 80 kb insertion between IGHV3-37 and IGHV7-40 highlighted in orange in Fig 5C. The insertion introduces extra copies of several gene fragments as annotated above. The region further inspected in panels (B) and (C) is highlighted in blue. (B) Alignment patterns of HiFi reads (rows) to GRCh38 (x axis) in the region highlighted in panel A. Grey bars denote aligned segments, with deletions and insertions denoted in red and purple respectively. Orange vertical lines indicate alignment breakpoints (i.e. alignments are clipped or split at these points). (C) Alignments of HiFi reads (rows) to HV31 (x axis) in the same region. The complex pattern of insertions, deletions and read clipping in panel (B) arise from between-copy misalignments that are largely resolved when aligning to HV31. (PNG) [file pcbi.1009254.s014.png]

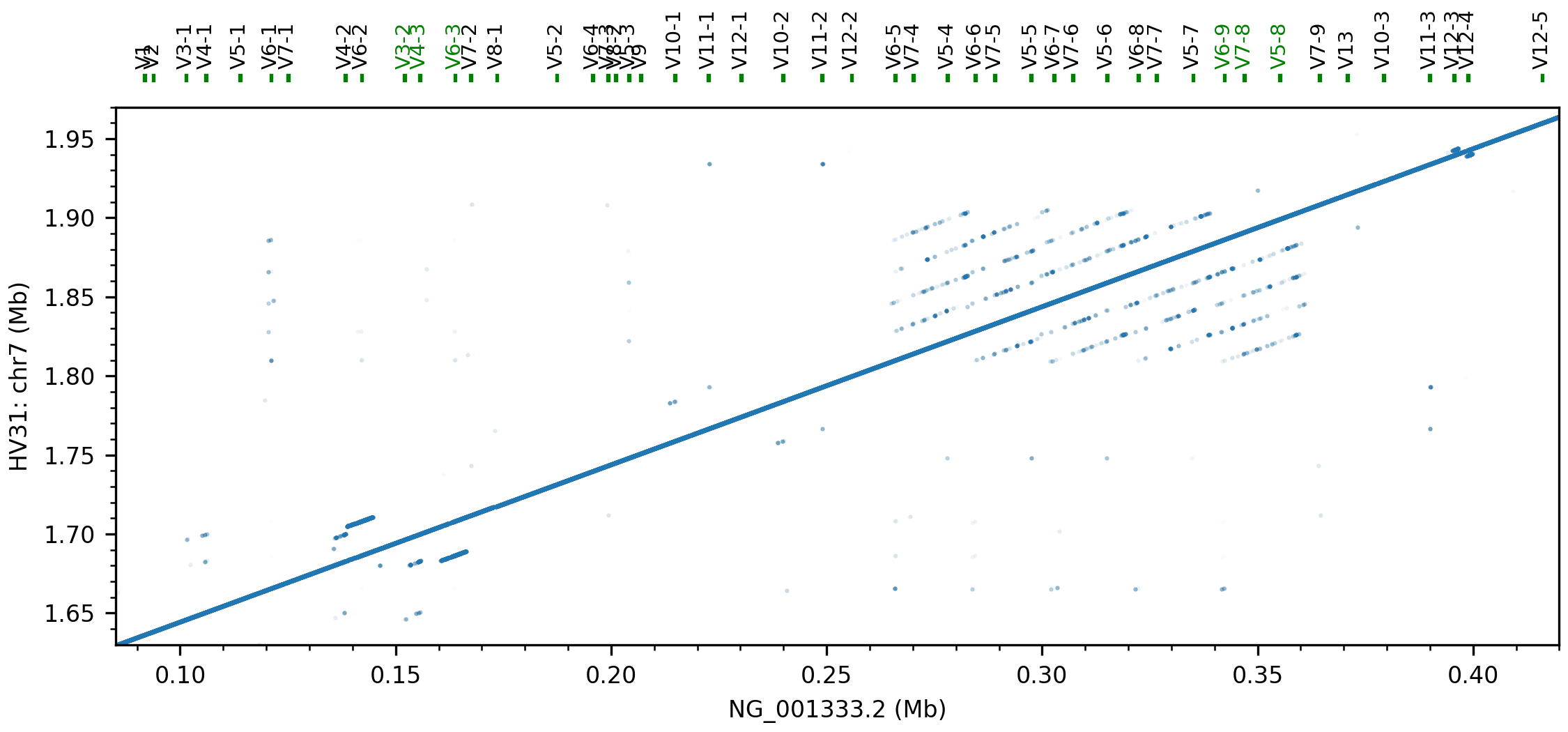

Supplement: S15 Fig — k-mer sharing plot (k = 50) comparing the HV31 assembly (y axis) with the NG_001333.2 contig from NCBI RefSeq (x axis). TRBV genes not included in GRCh38 are highlighted in green. (PNG) [file pcbi.1009254.s015.png]

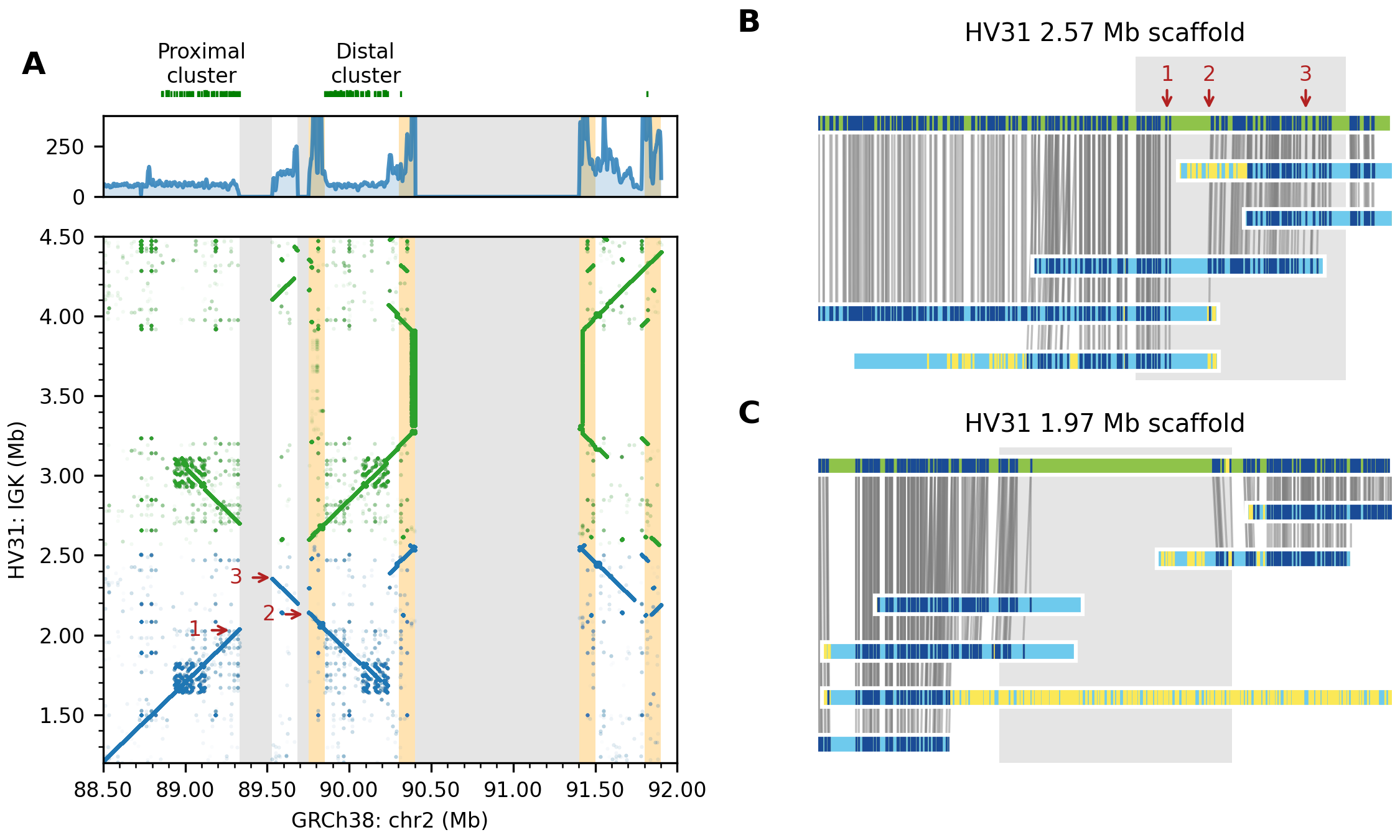

Supplement: S16 Fig — (A) k-mer sharing plot (k = 50) comparing GRCh38 (x axis) with the HV31 assembly (y axis). The 2.56 Mb scaffold and the 1.97 Mb scaffold in the HV31 assembly are shown in blue and green, respectively. Coverage of ONT reads aligned to GRCh38 is displayed above, and the proximal and distal clusters are annotated. Gaps in GRCh38 are shaded in gray. Novel sequence junctions in the HV31 assembly are annotated with red arrows. Sequence fragments of which extra copies were introduced in the HV31 assembly to fill in the gaps between IGK proximal and distal gene clusters in GRCh38 are highlighted in yellow; corresponding read coverage peaks confirm increased genome multiplicity of these fragments. (B) Alignment of Bionano contigs (blue) to the 2.56 Mb scaffold in the HV31 assembly (green). DLE-1 labels and their alignments are denoted by colored lines within and between scaffolds) as described in Fig 5D legend; note that all gray alignment lines connect the HV31 scaffold to each of the Bionano contigs (no between-Bionano alignments are shown). The approximate sequence region that maps to the GRCh38 gaps between IGK proximal and distal gene clusters is shaded in gray. For clarity, corresponding positions in the HV31 assembly in panels (A) and (B) are labelled with red arrows. (C) Alignment of BioNano contigs (blue) to the 1.97 Mb scaffold in the HV31 assembly (green). Approximate sequence region that maps to the GRCh38 heterochromatin gap is shaded in gray. (PNG) [file pcbi.1009254.s016.png]

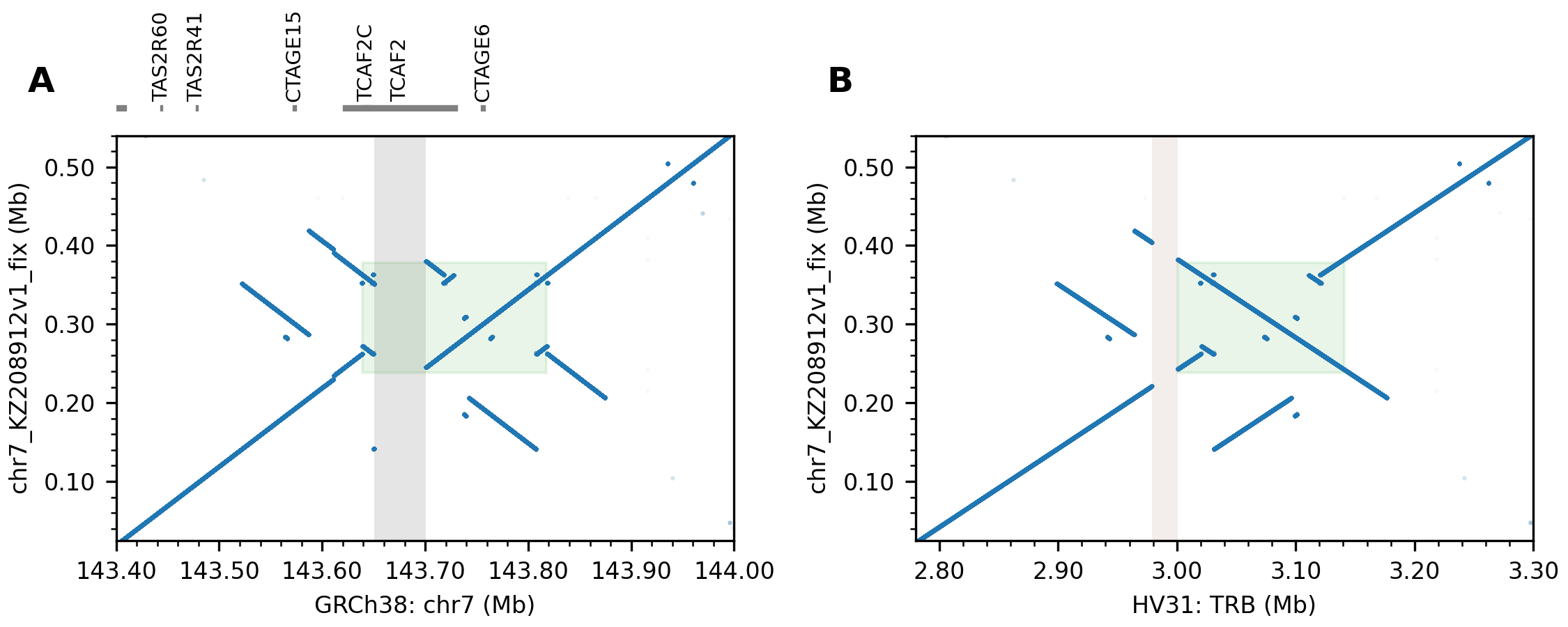

Supplement: S17 Fig — (A) k-mer sharing plot (k = 50) comparing the chr7_KZ208912v1_fix patch sequence (y axis) with GRCh38 (x axis), highlighting the genomic position corresponding to the 140 kb inversion in the HV31 assembly (green region) and a 50 kb gap in GRCh38 (gray region) which is closed in the HV31 assembly. (B) The HV31 assembly (x axis) is consistent with chr7_KZ208912v1_fix sequence (y axis) except for the 21.9 kb gap (brown) and the 140 kb inversion (green). (PNG) [file pcbi.1009254.s017.png]

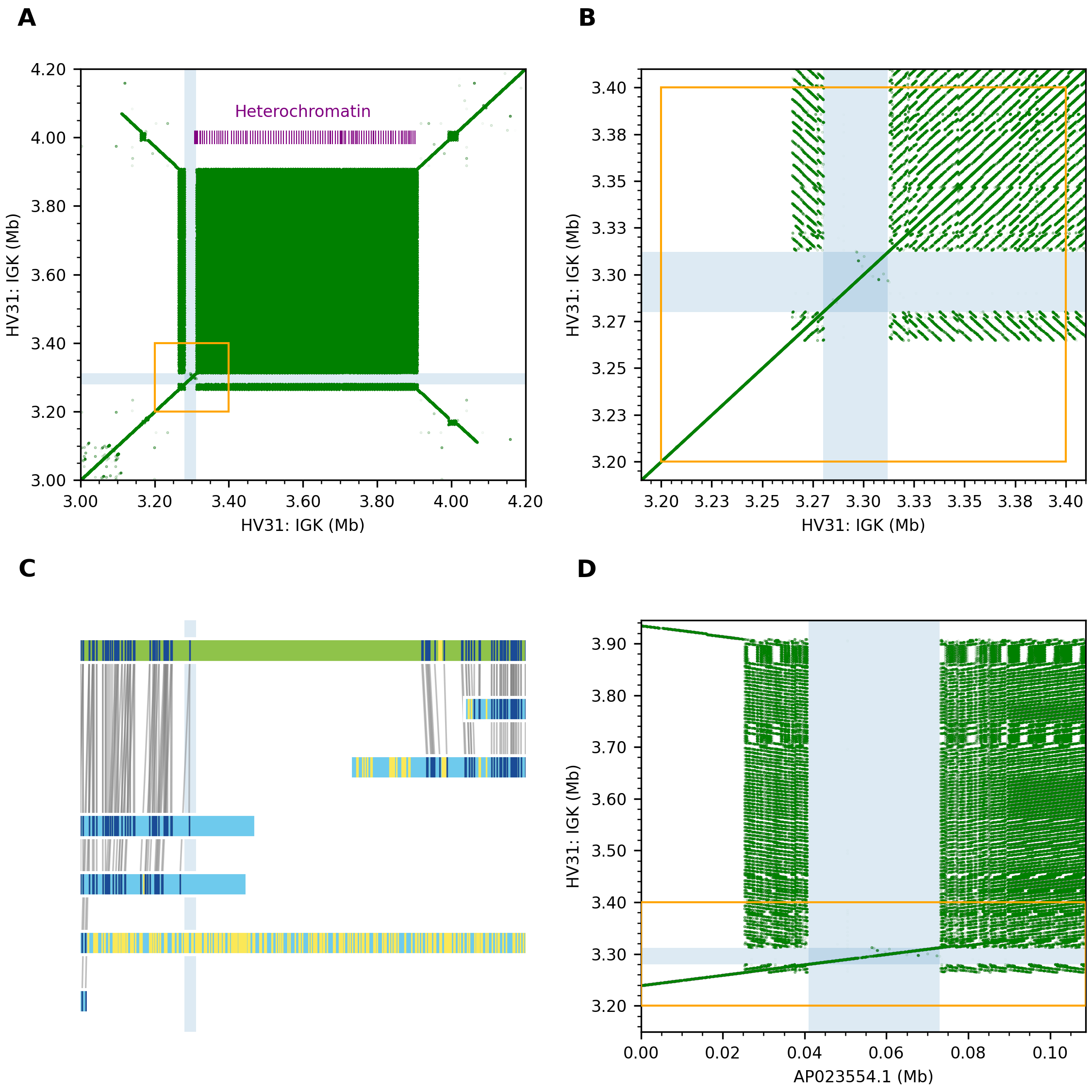

Supplement: S18 Fig — (A) k-mer sharing plot (k = 50) comparing the HV31 assembly with itself in the IGK heterochromatin region. Purple lines show the occurrence of a 22 bp HSat2B repeat signature sequence (TTCGATTCCATTTGATGATTCCAT). A 32 kb unique sequence fragment is highlighted in blue. (B) Details of k-mer sharing plot in panel (A), zoomed to reveal details of the unique sequence fragment and repeat structure. (C) Comparison of HV31 contigs and Bionano contigs as in panel C in S16 Fig, zoomed in to show that the 32 kb unique fragment (blue shaded region) contains a DLE-1 recognition label that was confirmed by Bionano contigs. (D) k-mer sharing plot (k = 50) comparing the HV31 assembly (y axis) with the GenBank AP023554.1 contig (x axis). For reference, the orange box in panels A, B and D denote approximately the same region. (PNG) [file pcbi.1009254.s018.png]

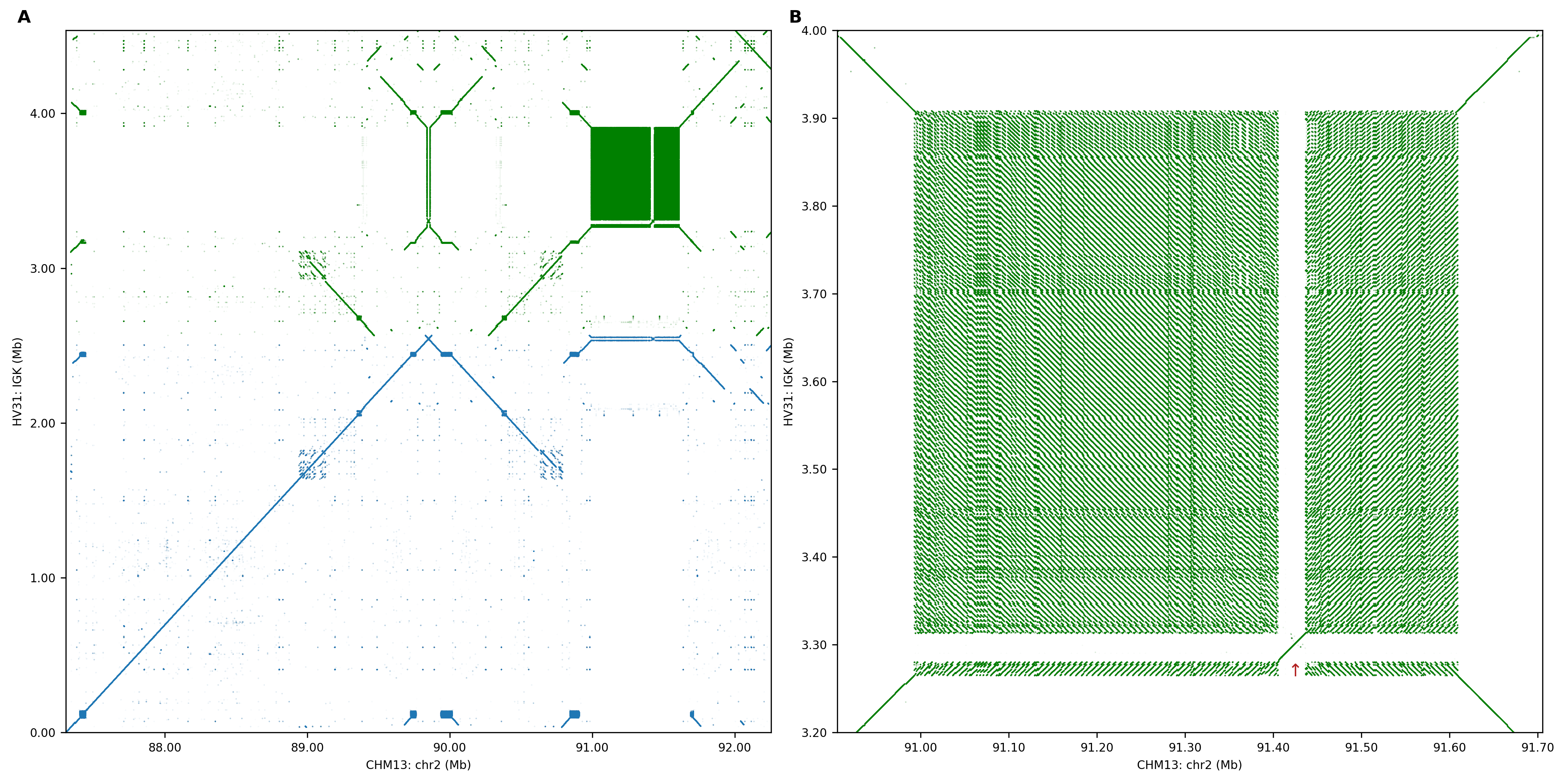

Supplement: S19 Fig — (A) k-mer sharing plot (k = 50) comparing the HV31 assembly (y axis) with the T2T CHM13 assembly (x axis) in the IGK region. The 2.56 Mb scaffold and the 1.97 Mb scaffold in the HV31 assembly are shown in blue and green, respectively. (B) k-mer sharing plot as in panel (A), zoomed in to show details of the heterochromatin region. The assemblies contain similar sequence in the heterochromatin region, but with some differences including in the position of the unique sequence (highlighted with a red arrow) as noted in main text. (PNG) [file pcbi.1009254.s019.png]

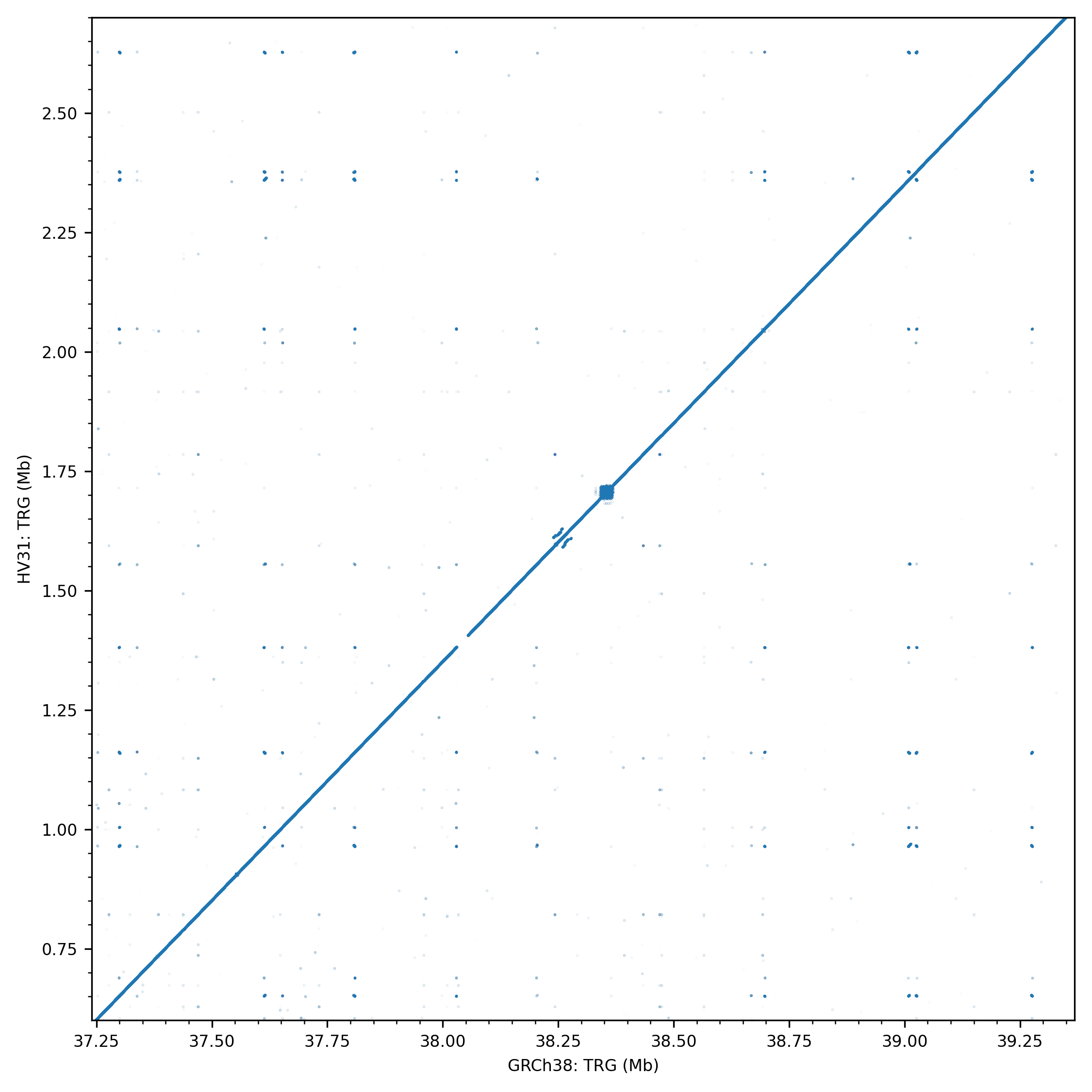

Supplement: S4 Dataset — Plots provide further detail of regional k-mer sharing plots shown in Fig 2, with details as described in Fig 2 legend and main text. In the IGK and IGL regions, colors reflect the distinct HV31 scaffolds. (ZIP) [file pcbi.1009254.s027.zip › k-mer sharing plots/TRG.png]

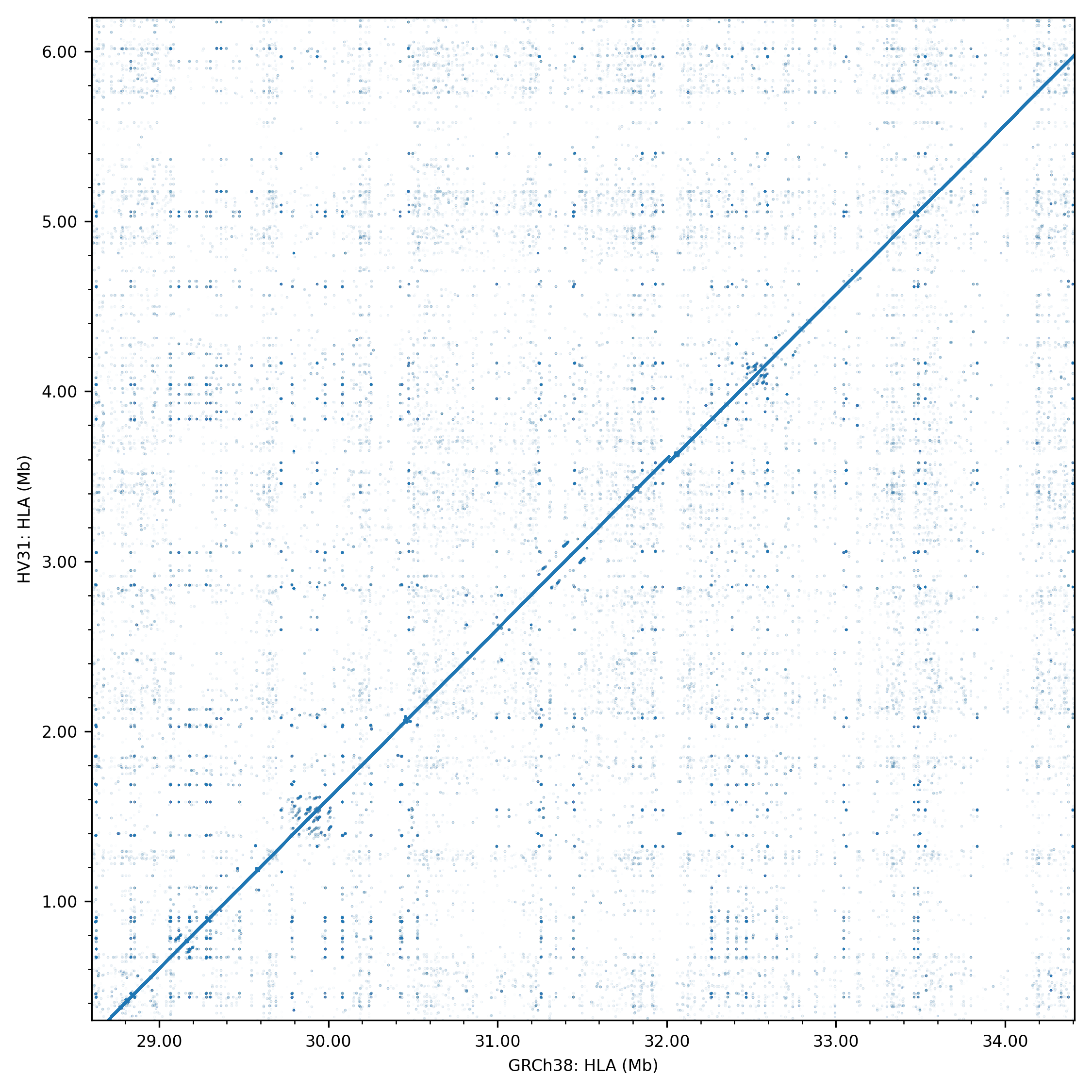

Supplement: S4 Dataset — Plots provide further detail of regional k-mer sharing plots shown in Fig 2, with details as described in Fig 2 legend and main text. In the IGK and IGL regions, colors reflect the distinct HV31 scaffolds. (ZIP) [file pcbi.1009254.s027.zip › k-mer sharing plots/HLA.png]

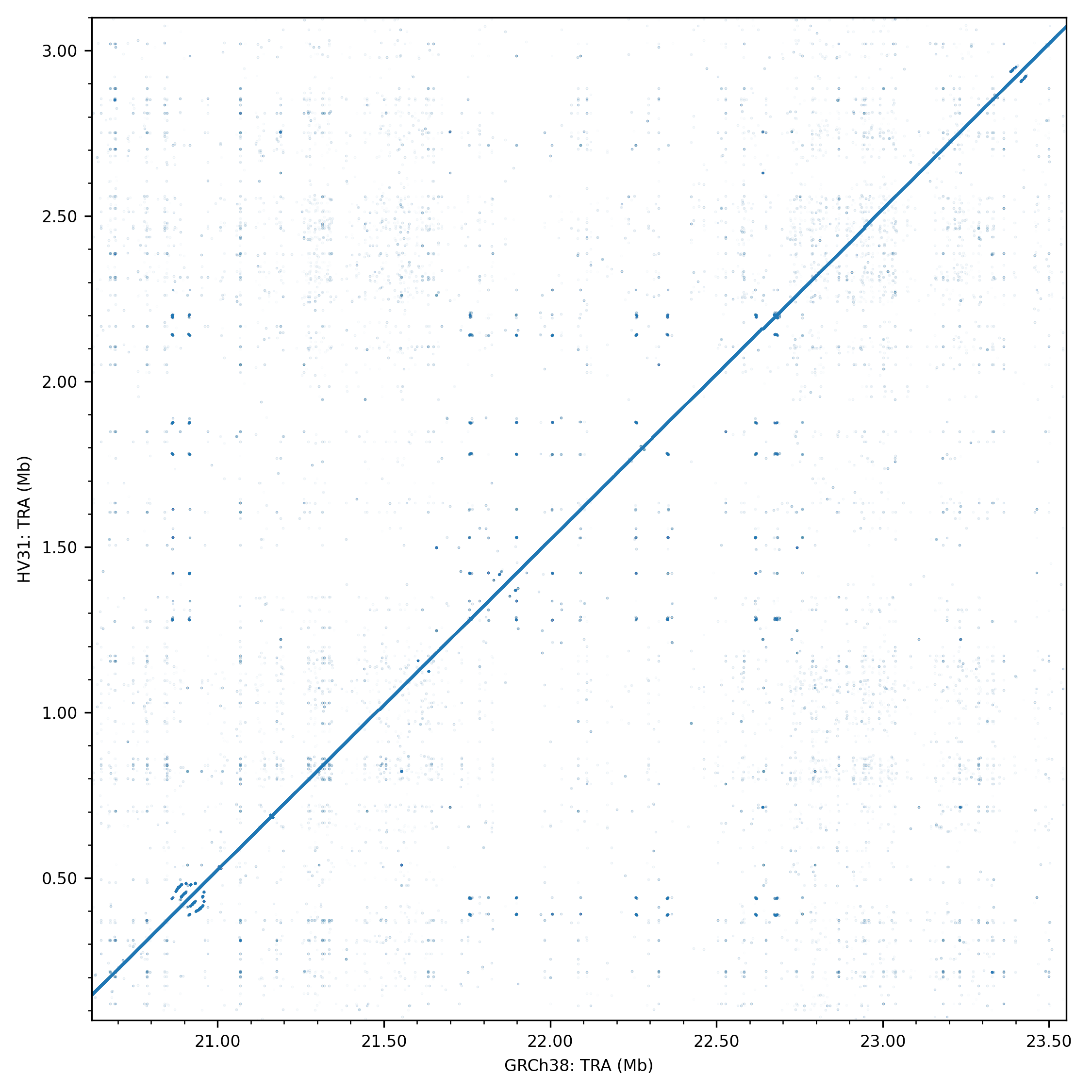

Supplement: S4 Dataset — Plots provide further detail of regional k-mer sharing plots shown in Fig 2, with details as described in Fig 2 legend and main text. In the IGK and IGL regions, colors reflect the distinct HV31 scaffolds. (ZIP) [file pcbi.1009254.s027.zip › k-mer sharing plots/TRA.png]

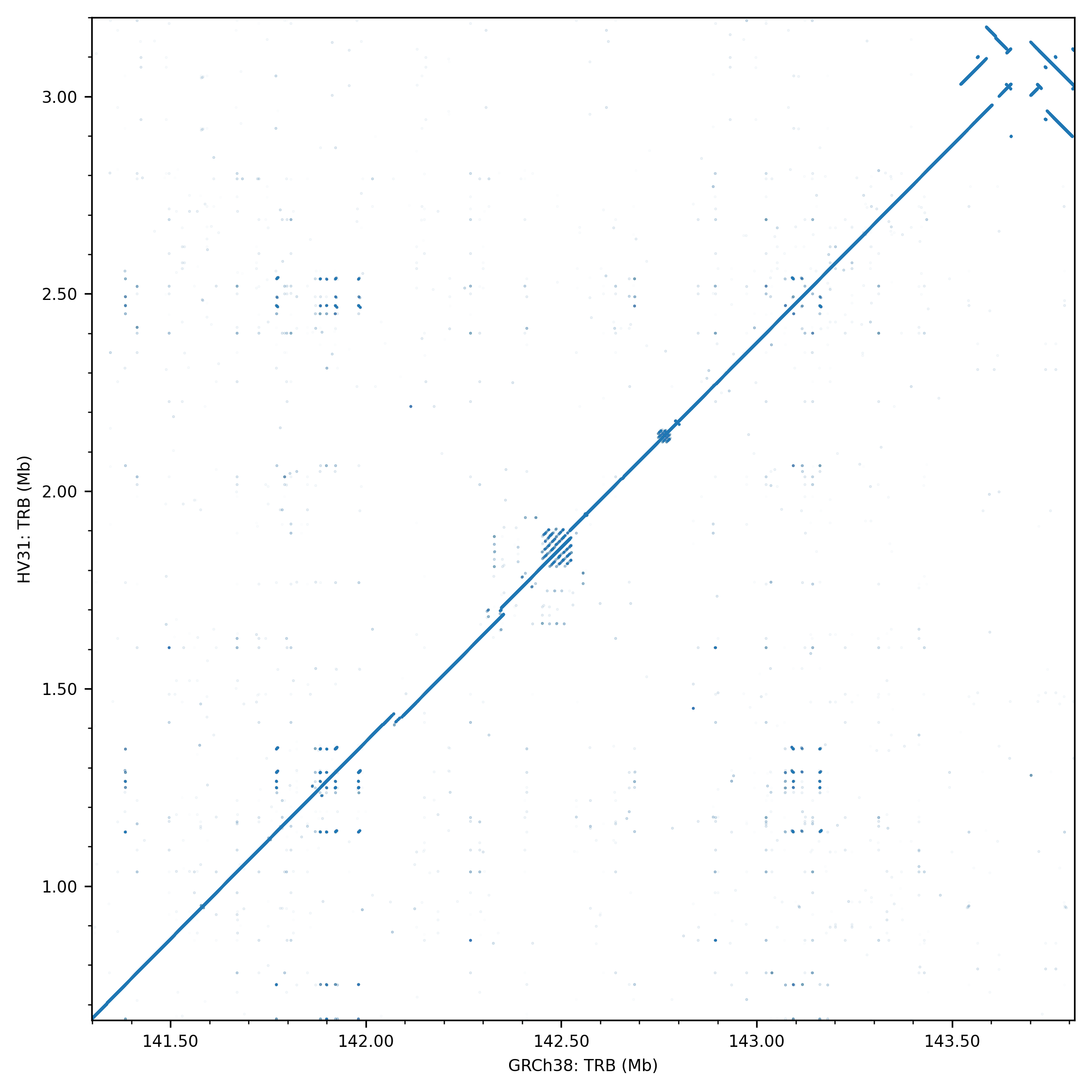

Supplement: S4 Dataset — Plots provide further detail of regional k-mer sharing plots shown in Fig 2, with details as described in Fig 2 legend and main text. In the IGK and IGL regions, colors reflect the distinct HV31 scaffolds. (ZIP) [file pcbi.1009254.s027.zip › k-mer sharing plots/TRB.png]

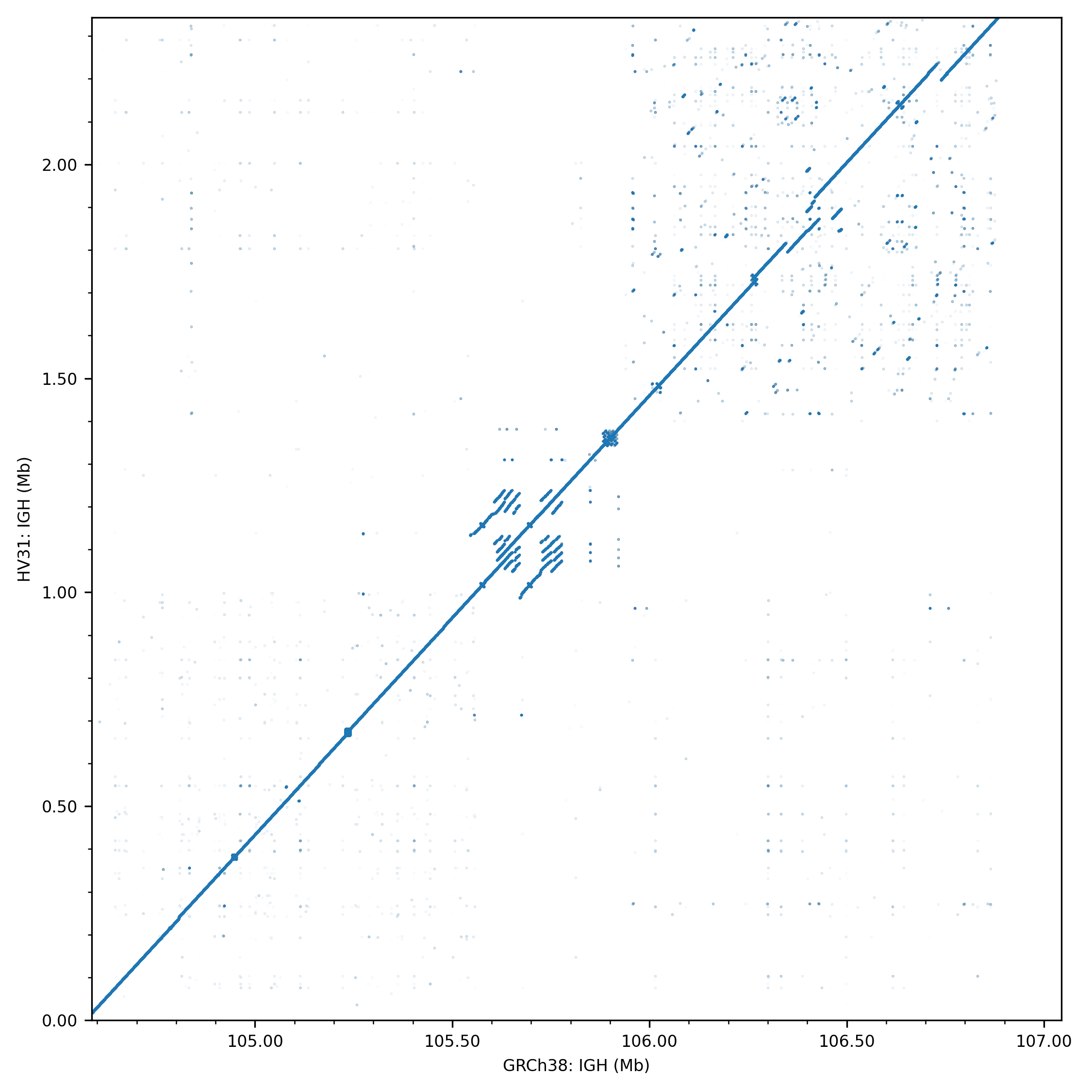

Supplement: S4 Dataset — Plots provide further detail of regional k-mer sharing plots shown in Fig 2, with details as described in Fig 2 legend and main text. In the IGK and IGL regions, colors reflect the distinct HV31 scaffolds. (ZIP) [file pcbi.1009254.s027.zip › k-mer sharing plots/IGH.png]

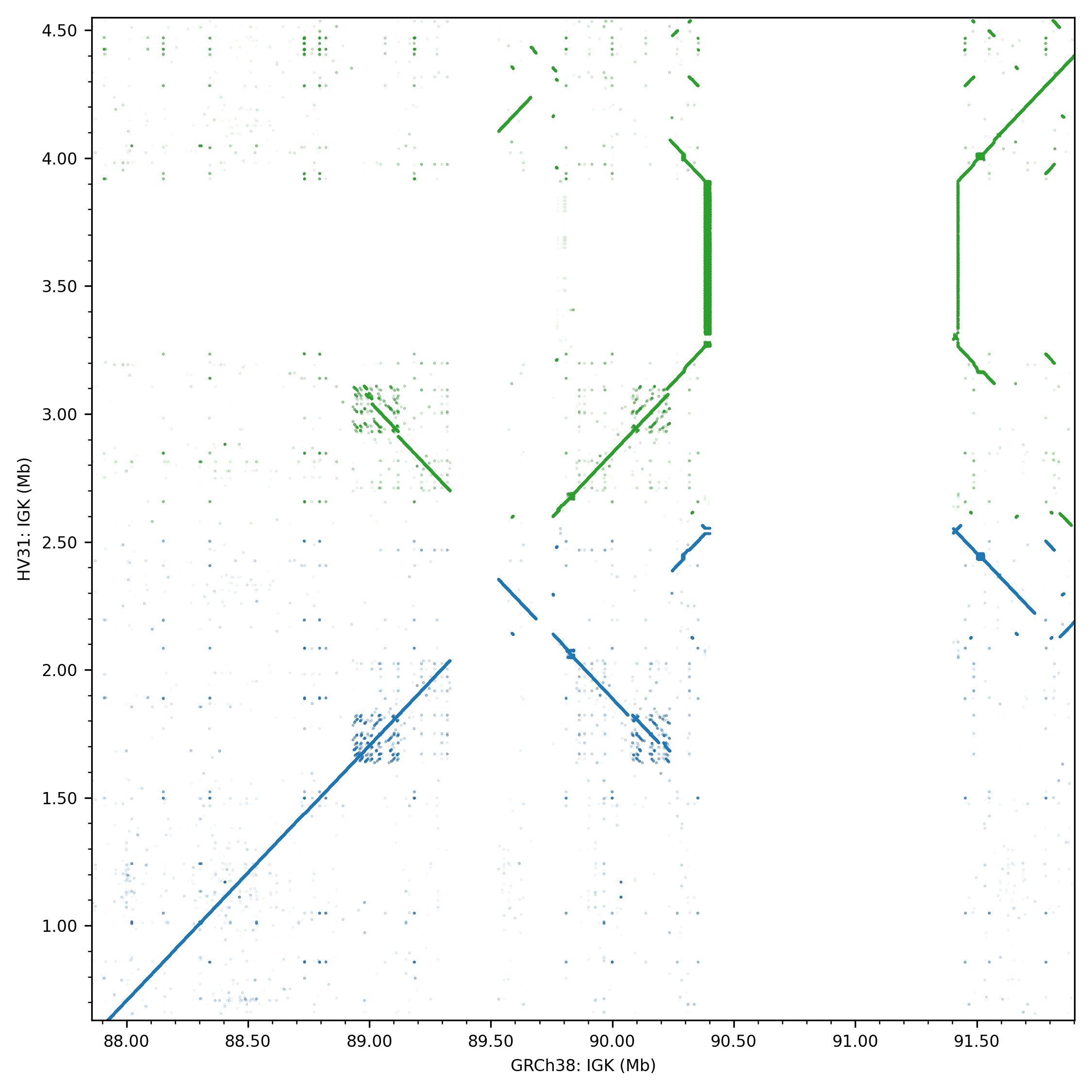

Supplement: S4 Dataset — Plots provide further detail of regional k-mer sharing plots shown in Fig 2, with details as described in Fig 2 legend and main text. In the IGK and IGL regions, colors reflect the distinct HV31 scaffolds. (ZIP) [file pcbi.1009254.s027.zip › k-mer sharing plots/IGK.png]

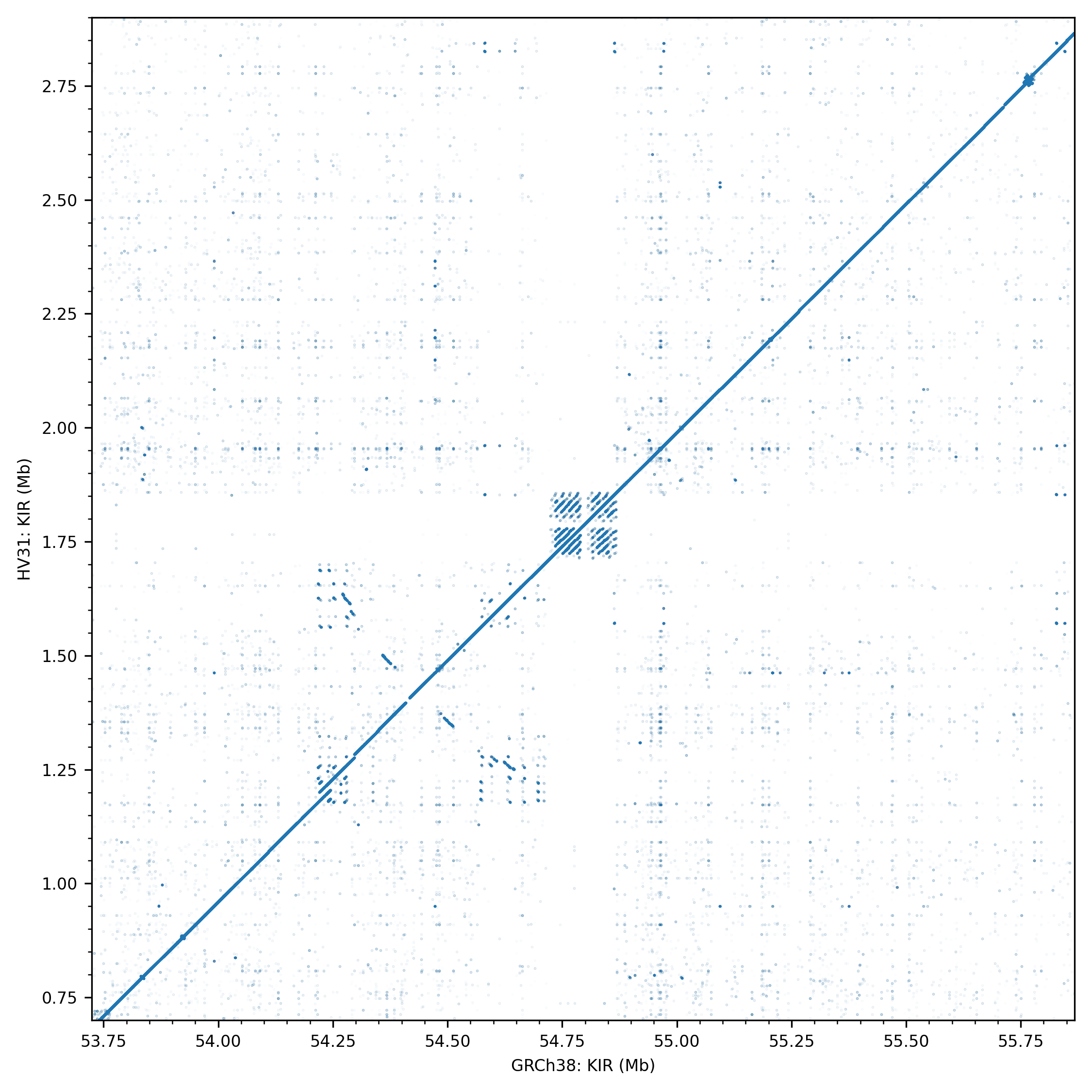

Supplement: S4 Dataset — Plots provide further detail of regional k-mer sharing plots shown in Fig 2, with details as described in Fig 2 legend and main text. In the IGK and IGL regions, colors reflect the distinct HV31 scaffolds. (ZIP) [file pcbi.1009254.s027.zip › k-mer sharing plots/KIR.png]

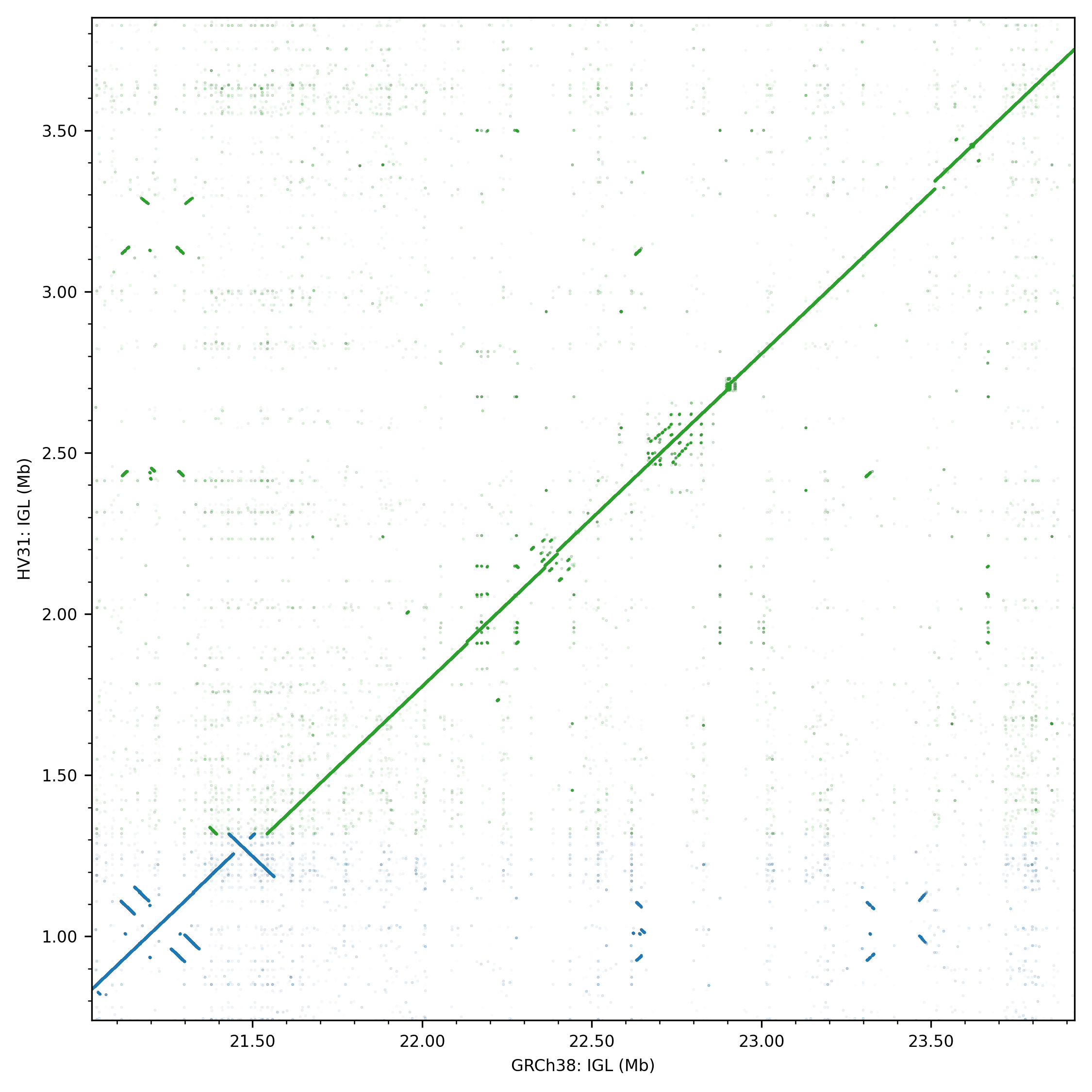

Supplement: S4 Dataset — Plots provide further detail of regional k-mer sharing plots shown in Fig 2, with details as described in Fig 2 legend and main text. In the IGK and IGL regions, colors reflect the distinct HV31 scaffolds. (ZIP) [file pcbi.1009254.s027.zip › k-mer sharing plots/IGL.png]
