# Supplementary material for: Using de novo assembly to identify structural variation of eight complex immune system gene regions
Source: PLoS Comput Biol. 2021 Aug 3;17(8):e1009254. doi: 10.1371/journal.pcbi.1009254 (PMC8363018; doi:10.1371/journal.pcbi.1009254)
Supplement: S1 Table — Table shows the name, cell type, genome coverage estimated by alignments to GRCh38, and read length statistics for each dataset generated in this study. (PDF) [file pcbi.1009254.s020.pdf]

**S1 Table**

| Name | Cell type | Coverage | Read length |
|---|---|---|---|
| PacBio Circular Consensus Sequencing (CCS) | $CD14^+$ monocytes | 12.3× | Mean = 12.7 kb; N50 = 12.7 kb |
| PacBio Continuous Long Reads (CLR) | $CD14^+$ monocytes | 35× | Mean = 15.4 kb; N50 = 25.9 kb |
| Oxford Nanopore PromethION (ONT) | $CD14^+$ monocytes | 63× | Mean = 8.7 kb; N50 = 10.9 kb |
| Bionano DLS optical mapping | PBMC | 152.7× | Mean = 149.4 kb; N50 = 216.4 kb |
| MGI standard short-read sequencing | PBMC | 56.8× | 100 bp paired-end |
| MGI coolMPS sequencing | PBMC | 56.9× | 100 bp paired-end |
| MGI stLFR linked-read sequencing | $CD14^+$ monocytes | 51.3× | 100 bp paired-end |
| 10X Linked-Read sequencing | $CD14^+$ monocytes | 40.2× | 150 bp paired-end |
| Illumina PCR-free sequencing | PBMC | 44.2× | 151 bp paired-end |
