# Supplementary material for: Using de novo assembly to identify structural variation of eight complex immune system gene regions
Source: PLoS Comput Biol. 2021 Aug 3;17(8):e1009254. doi: 10.1371/journal.pcbi.1009254 (PMC8363018; doi:10.1371/journal.pcbi.1009254)
Supplement: S2 Table — Table shows per-base error rate estimates for local scaffolds and the finished HV31 assembly (Fig 1A) using the Merqury algorithm, and the modified algorithm described in Methods and Fig 1D legend. As described in Methods, the key difference is that we estimate based on clusters of kmers with low validation coverage and this produces slightly higher estimates in practice than the Merqury method. (PDF) [file pcbi.1009254.s021.pdf]

S2 Table

	Merqury		This work	
**Region**	*Before polishing*	*After polishing*	*Before polishing*	*After polishing*
IGH	88.5	6.7	86.6	8.1
IGK	223.2	24.2	183.0	42.3
IGL	78.8	8.0	68.2	11.9
HLA	32.4	3.3	39.5	5.1
TRA	71.7	18.9	85.7	22.8
TRB	62.6	7.4	52.2	9.6
TRG	76.6	18.1	86.0	21.2
KIR	78.1	18.1	94.2	25.0

Unit: errors per Mb
