# Supplementary material for: Using de novo assembly to identify structural variation of eight complex immune system gene regions
Source: PLoS Comput Biol. 2021 Aug 3;17(8):e1009254. doi: 10.1371/journal.pcbi.1009254 (PMC8363018; doi:10.1371/journal.pcbi.1009254)
Supplement: S3 Table — Table shows details of locations where validation k-mers show discrepancy from expectation (as numbered in S10 Fig) and our conclusion about the region. For each region, the table lists the relevant assembled region (Table 1), number as shown in S10 Fig, type of evidence we have inspected to provide additional evidence, and our conclusion about the cause of discrepancy. (PDF) [file pcbi.1009254.s022.pdf]

S3 Table

Region	Number	Evidence	Speculation
IGH	1	CLR reads	Misalignment.
IGH	2	CCS reads	Heterozygous deletion.
IGH	3	CLR reads	Heterozygous duplication; see S8 Fig.
IGK	4	HV31 assembly	Assembly gap.
IGK	5	Bionano contigs and CCS reads	Misalignment.
IGK	6	-	Inconclusive.
IGK	7	-	Inconclusive.
IGK	8	-	Inconclusive.
IGK	9	ONT reads	Heterochromatin microsatellite array; see S12 Fig
IGK	10	-	Inconclusive.
IGL	11	Bionano contigs	Misalignment; possible assembly error outside the IGL region.
IGL	12	Bionano contigs	Heterozygous deletion.
HLA	13	CCS reads	Heterozygous deletion; see S4 Fig.
HLA	14	CCS reads	Assembly error (collapsed duplications); see S4 Fig.
TRB	15	Bionano contigs and CCS reads	Heterozygous duplication
TRB	16	CLR reads	Misalignment.
TRB	17	HV31 assembly	Assembly gap.
TRB	18	Bionano contigs and HV31 assembly	Misalignment due to assembly gap (see number 17).
TRG	19	HV31 assembly	Assembly gap.
KIR	20	HV31 assembly	Assembly gap.
