# Supplementary material for: Using de novo assembly to identify structural variation of eight complex immune system gene regions
Source: PLoS Comput Biol. 2021 Aug 3;17(8):e1009254. doi: 10.1371/journal.pcbi.1009254 (PMC8363018; doi:10.1371/journal.pcbi.1009254)
Supplement: S4 Table — (PDF) [file pcbi.1009254.s023.pdf]

S4 Table

Name	Version	Reference
Assemblytics	1.2.1	[1]
Bionano Solve	3.5.1_01142020	[2]
Canu	1.9	[3]
Jellyfish	2.3.0	[4]
Long Ranger	2.2.2	[5]
Minimap2	2.14	[6]
MUMmer	4.0.0beta2	[7]
Peregrine	0.1.6.1	[8]
Pilon	1.23	[9]
Samtools	1.1	[10]
PBSV	2.2.1	[11]
Sniffles	1.0.12a	[12]
SVanalyzer (SVmerge)	0.36	[13]
TGS-GapCloser	1.1.1	[14]

References

1. Nattestad M, Schatz MC. Assemblytics: a web analytics tool for the detection of variants from an assembly. Bioinformatics. 2016;32: 3021–3023. doi:10.1093/bioinformatics/btw369
2. Bionano Genomics. Software Downloads. Available: https://bionanogenomics.com/support/software-downloads/
3. Koren S, Walenz BP, Berlin K, Miller JR, Bergman NH, Phillippy AM. Canu: scalable and accurate long-read assembly via adaptive *k* -mer weighting and repeat separation. Genome Res. 2017;27: 722–736. doi:10.1101/gr.215087.116
4. Marçais G, Kingsford C. A fast, lock-free approach for efficient parallel counting of occurrences of k-mers. Bioinformatics. 2011;27: 764–770.
5. 10x Genomics. What is Long Ranger? Available: https://support.10xgenomics.com/genome-exome/software/pipelines/latest/what-is-long-ranger
6. Li H. Minimap2: pairwise alignment for nucleotide sequences. Bioinformatics. 2018;34: 3094–3100.
7. Delcher AL, Phillippy A, Carlton J, Salzberg SL. Fast algorithms for large-scale genome alignment and comparison. Nucleic Acids Res. 2002;30: 2478–2483.
8. Chin C-S, Khalak A. Human Genome Assembly in 100 Minutes. BioRxiv; 2019 Jul. doi:10.1101/705616
9. Walker BJ, Abeel T, Shea T, Priest M, Abouelliel A, Sakthikumar S, et al. Pilon: An Integrated Tool for Comprehensive Microbial Variant Detection and Genome Assembly Improvement. Wang J, editor. PLoS ONE. 2014;9: e112963. doi:10.1371/journal.pone.0112963
10. Li H, Handsaker B, Wysoker A, Fennell T, Ruan J, Homer N, et al. The sequence alignment/map format and SAMtools. Bioinformatics. 2009;25: 2078–2079.
11. PacificBiosciences. pbsv: PacBio structural variant (SV) calling and analysis tools. In: Github [Internet]. Available: https://github.com/PacificBiosciences/pbsv
12. Sedlazeck FJ, Rescheneder P, Smolka M, Fang H, Nattestad M, von Haeseler A, et al. Accurate detection of complex structural variations using single-molecule sequencing. Nat Methods. 2018;15: 461–468. doi:10.1038/s41592-018-0001-7
13. Zook JM, Hansen NF, Olson ND, Chapman L, Mullikin JC, Xiao C, et al. A robust benchmark for detection of germline large deletions and insertions. Nat Biotechnol. 2020;38: 1347–1355. doi:10.1038/s41587-020-0538-8
14. Xu M, Guo L, Gu S, Wang O, Zhang R, Fan G, et al. TGS-GapCloser: fast and accurately passing through the Bermuda in large genome using error-prone third-generation long reads. bioRxiv. 2019; 831248.
